# Differential inflammation, oxidative stress and cardiovascular damage markers of nano- and micro-particle exposure in mice: Implications for human disease burden

**DOI:** 10.1016/j.redox.2025.103644

**Published:** 2025-04-22

**Authors:** Marin Kuntic, Ivana Kuntic, Dirk Cleppien, Andrea Pozzer, David Nußbaum, Matthias Oelze, Tristan Junglas, Lea Strohm, Henning Ubbens, Steffen Daub, Maria Teresa Bayo Jimenez, Sven Danckwardt, Thomas Berkemeier, Omar Hahad, Matthias Kohl, Sebastian Steven, Albrecht Stroh, Jos Lelieveld, Thomas Münzel, Andreas Daiber

**Affiliations:** a University Medical Center Mainz, Department for Cardiology 1, Molecular Cardiology, Mainz, Germany; b German Center for Cardiovascular Research (DZHK), Partner Site Rhine-Main, Mainz, Germany; c Leibniz Institute for Resilience Research, Mainz, Germany; d Max Planck Institute for Chemistry, Atmospheric Chemistry Department, Mainz, Germany; e Center for Thrombosis and Hemostasis (CTH), University Medical Center of the Johannes Gutenberg-University, Mainz, Germany; f University Medical Center Ulm, Department of Clinical Chemistry, Ulm, Germany; g Max Planck Institute for Chemistry, Multiphase Chemistry Department, Mainz, Germany; h Division of Cardiology, Goethe University Frankfurt, University Hospital, Department of Medicine III, Frankfurt a. M., Germany; i University Medical Center Mainz, Institute of Pathophysiology, Mainz, Germany; j Institute of Physiology I, University Hospital Muenster, Germany

**Keywords:** Size-dependent effects particulate matter, Oxidative stress, Inflammation, Ultrafine particle exposure, Cardiovascular disease

## Abstract

Particulate matter (PM) poses a significant risk to human health; however, it remains uncertain which size fraction is especially harmful and what mechanisms are involved. We investigated the varying effects of particle size on specific organ systems using a custom mouse exposure system and synthetic PM (SPM). Whole-body exposure of mice showed that micrometer-sized fine SPM (2–4 μm) accumulated in the lungs, the primary entry organ, while nanometer-sized SPM (<250 nm) did not accumulate, suggesting a transition into circulation. Mice exposed to micro-SPM exhibited inflammation and NADPH oxidase-derived oxidative stress in the lungs. In contrast, nano-SPM-exposed mice did not display oxidative stress in the lungs but rather at the brain, heart, and vascular levels, supporting the hypothesis that they penetrate the lungs and reach the circulation. Sources of reactive oxygen species from micro-SPM in the lung are NOX1 and NOX2, driven by pulmonary inflammation, while oxidative stress from nano-SPM in the heart is mediated by protein kinase C-dependent p47^phox^ phosphorylation, leading to NOX2 activation in infiltrated monocytes. Endothelial dysfunction and increased blood pressure were more pronounced in nano-SPM-exposed mice, also supported by elevated endothelin-1 and reduced endothelial nitric oxide synthase expression, which enhances constriction and diminishes vasodilation. Further, we estimated the cardiovascular disease burden of nano-particles in humans based on global exposure data and hazard ratios from an epidemiological cohort study. These results provide novel insights into the disease burdens of inhaled nano- and micro-particles (corresponding to fine and ultrafine categories), guiding future studies.

## Highlights

• Particulate matter from air pollution contributes primarily by cardiovascular sequelae to the global burden of disease.
• Contrary to fine (μm-sized) particles, ultrafine (nm-sized) particles' health effects and pathomechanisms are understudied.
• In mice, micro-particles affect the lungs, while nano-particles cause remote organ damage by oxidative stress/inflammation.
• In humans, ultrafine particles account for a significant part of the cardiovascular disease burden by air pollution.
• Therefore, ultrafine particles should be more extensively considered in clinical and epidemiological studies.

## Graphical abstract

**Figure:**
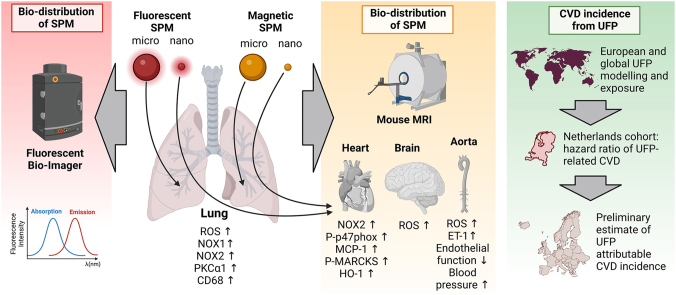

## 1 Introduction

Air pollution is a major contributor to non-communicable diseases [1]. The Global Burden of Disease study ranks air pollution as the foremost risk factor for disability-adjusted life years (DALYs) and deaths [2,3], with annual excess mortality estimates ranging from around eight million [4,5] to ten million [6]. Particulate matter (PM), a leading component of air pollution, is particularly harmful, impacting the cardiovascular system [7]. PM includes all solid and liquid particles in the air [8,9] and varies in sources, structure, composition, and size. The composition of PM is determined by its origin (natural or anthropogenic) and environmental interactions [10,11]. PM is commonly classified by diameter: PM_10_ (<10 μm), PM_2.5_ (<2.5 μm), and ultra-fine (or nano-) particles (UFPs, <0.1 μm) [12], with most health studies and regulations focusing on the mass concentrations of PM_10_ and PM_2.5_ [13]. Importantly, PM_2.5_ encompass nanoparticles by definition. The major sources of UFP are combustion (burning liquid fuels in engines and solid fuels for heating), tire and brake wear, and catalytic converters. All these sources contribute to the particle size distribution, including UFP and “nano-sized” PM [14]. Micrometer-sized PM additionally originates from natural sources like dust or forest fires, but also arises due to coagulation and growth of smaller PM.

Exposure to urban particles leads to cardiovascular and other end-organ damage, largely mediated by activation and infiltration of immune cells and reactive oxygen species (ROS) formation by the phagocytic NADPH oxidase, NOX2, demonstrated by genetic deletion of key components such as Toll-like receptor-4 and gp91^phox^ in this process [15]. PM_2.5_ exposure also promotes atherosclerotic plaque formation through oxidative stress mechanisms in mice [16] and humans [17], as well as acute myocardial infarction due to high peak concentrations of PM [18]. Specifically, UFPs can reach the mitochondrial matrix, causing disturbances in mitochondrial respiration and the generation of superoxide radicals and hydrogen peroxide, contributing to damage in surrounding tissues [9].

Ambient PM typically has a mineral or carbon core and carries chemicals like inorganic salts, organic compounds, transition and heavy metals, and endotoxins [10,19,20]. The ability of PM to induce oxidative stress *in vivo* is directly connected to its oxidative potential, largely determined by particle loading with redox-active chemicals (metals or redox-cycling organic compounds) or natural pyrogens that can activate immune cells, leading to oxidative burst [[21], [22], [23]]. The oxidative potential is usually determined by acellular assays (e.g. oxidation kinetics of dithiothreitol) [24]. The oxidative potential is inversely associated with markers of cardiovascular health, such as microvascular function [25] and is likely positively associated with the circulating number of white blood cells [26]. Exposure of mice to ambient particles with high oxidative potential also caused impaired microvascular function of cerebral and retinal arterioles [27].

Measuring PM size and number concentrations is relatively straightforward and less challenging than differentiating composition, especially for particles in the PM_2.5_ and PM_10_ size ranges [28]. Accordingly, PM_2.5_ and PM_10_ mass concentrations (in μg/m^3^) are widely adopted parameters in health studies and regulations, while UFP mass and number concentrations are less commonly measured and used [29,30]. Smaller particles tend to be more harmful than larger ones, and research often prioritizes PM_2.5_ over PM_10_ [12,31]. UFPs may be especially detrimental, likely due to their ability to penetrate the air-blood barrier [32,33]. While UFPs dominate ambient particle size distributions by number, they only comprise a small fraction of PM_2.5_ mass [34]. Therefore, using PM_2.5_ or PM_10_ mass concentrations as a predictor for health endpoints not only disregards the composition of particles but also the differential harmfulness of particle sizes within PM. While smaller particles contribute comparatively little to mass concentrations, they may be disproportionately more harmful. While previous clinical studies generally overlooked UFP health effects, recent technological innovations and more affordable devices now enable UFP measurement more broadly.

This study aimed to determine if ultrafine or nano-particles penetrate the air-blood barrier more readily than microparticles and if they can be detected in remote organs. We also sought reliable functional and molecular markers related to cardiovascular and pulmonary systems to differentiate the level of harm and tissue transmigration between particle sizes. Hazard ratios from a cohort were used to estimate UFP effects on cardiovascular disease incidence in humans, highlighting their importance in disease outcomes.

## 2 Materials and methods

### 2.1 Exposure of laboratory animals

All animals were treated following the Guide for the Care and Use of Laboratory Animals as adopted by the US National Institutes of Health, and approval was granted by the Ethics Committee of the University Medical Center Mainz and the Landesuntersuchungsamt Rheinland-Pfalz (Koblenz, Germany; permit number: 23,177-07/G 20-1-055). All mice were housed under a 12-h light/dark cycle in the ventilated animal cabinet and fed ad libitum. Male C57BL/6 mice, 8–12 weeks old, were exposed to either fluorescent or magnetic particles of two different sizes each or fresh air. Exposures to fresh air and one particle size were always performed on the same day using littermate mice from the same shipment. The exposure lasted for 6 h per day for 3 days. The average concentration of all synthetic particulate matter (SPM) in the exposure chamber was 230 ± 46 μg/m^3^.

The PM concentration range was chosen because 200–300 μg/m^3^ is a peak concentration reached in the most polluted cities [35,36]. The relation between mouse exposure and human exposure is approximately similar. Mice have a respiratory rate of 80–230 min^−1^ [37] and tidal volume of 0.2 mL [38], making the total mass of PM being inhaled during a 6 h exposure session (approximate chamber concentration of 200 μg/m^3^) 1.15–3.31 μg (assuming 100 % PM retention). Assuming the mouse weight of 25 g, the 6 h exposure session results in 46–132 μg/kg/day. The human respiratory rate is approximately 10–20 min^−1^ and the tidal volume is approximately 0.5 L, and assuming a body mass of 60 kg and the same exposure of 200 μg/m^3^, a human would inhale 24–48 μg/kg/day. Mouse exposure occurs during the sleeping phase when the respiratory activity is in the lower range, we can assume that mouse and human exposures are on par.

The custom exposure system (described in detail in Ref. [27]) was acquired from TSE Systems GmbH (Hochtaunuskreis, Germany). Fluorescent SPM were acquired from Spherotec (Lake Forest, IL, US), nano-SPM (FP-0256-2, Nile Red, 0.25 μm, polystyrene) and micro-SPM (FP-2065-2, Nile Blue, 2.16 μm, polystyrene). Magnetic SPM were acquired from Kisker Biotech GmbH (Steinfurt, Germany), nano-SPM (PMSI-H-0.25-5 (https://www.kisker-biotech.com/article/PMSI-H.25-5), superparamagnetic silica-encapsulated FeO_x_ particles, 0.25 μm), micro-SPM (PM-4.5 (https://www.kisker-biotech.com/article/PM-4.5), magnetic polystyrene-encapsulated FeO_x_ particles, 4.13 μm). The particles were suspended in CLRwater and placed in the collision nebulizer of the exposure system. After nebulizing into an aerosol, the particle suspension droplets passed through a drying column and dry particles entered the exposure chamber. The mass concentration of SPM was monitored by a particle detector that consists of two instruments for different particle size ranges combined into a NanoSpectroPan instrument (TSE Systems GmbH, Germany). The electric field mobility spectrometer measured the particles in the size range from 0 to 0.2 μm, and the light scattering detector measured in the 0.2–35 μm range. The measured values of the mean SPM mass concentration in the exposure chamber were: 248 ± 66 μg/m^3^ for nano-fluorescent SPM, 270 ± 65 μg/m^3^ for micro-fluorescent SPM, 209 ± 37 μg/m^3^ for nano-magnetic SPM and 221 ± 27 μg/m^3^ for micro-magnetic SPM.

After exposure, the mice were sacrificed by transection of the diaphragm and removal of the heart and thoracic aorta under deep ketamine/xylazine anesthesia (i.p. 120/16 mg/kg body weight), and tissues were harvested. The mouse exposure paradigm is shown in Suppl. Fig. S1.

### 2.2 Exposure model and emissions for human studies

We applied a data-informed global atmospheric modelling method to compute the exposure to air pollutants. The Earth system model with comprehensive atmospheric chemistry (EMAC) used in this study simulates the role of natural and anthropogenic emissions of trace gases and particles in atmospheric composition and exposure [39,40]. Anthropogenic sources were adopted from the Community Emission Data System (CEDS) and the Emissions Database for Global Atmospheric Research (EDGAR) [41,42]. Source sectors include fossil energy production, industry, land transport, shipping, aviation, domestic energy use from solid biofuels, waste incineration, agriculture, solvent production and use. A comprehensive evaluation of the modelled atmospheric dust, black and organic carbon, aerosol optical depth, and aerosol organic and inorganic compounds was presented previously [40,43,44]. Methods for downscaling UFP concentrations to a high spatial resolution and estimation of exposure and potential impacts on cardiovascular disease incidence are presented in the online supplement.

### 2.3 Additional methods are described in the online supplement

Isometric tension studies in isolated aortic rings to determine endothelial (vascular) function and non-invasive blood pressure measurement was used as previously described [45,46]. Using previously described methods, we detected fluorescent SPM in the isolated organs [47,48] and iron oxide SPM in the whole body via MRI [49,50]. Dihydroethidium fluorescence microtopography was used to determine aortic, pulmonary and cerebral ROS levels as previously described [27,45,46]. Western blot analysis was used to determine protein expression in different tissues as previously described [27,45,46]. Detailed description of these methods is available in the online supplement.

### 2.4 Statistics

Where possible, the results are presented as bar graphs with individual values. Two-way ANOVA (with Tukey's correction for comparison of multiple means) was used for comparisons of concentration-relaxation curves. One-way ANOVA (with Tukey's post-hock analysis for comparison of multiple means) was used for comparisons of all other data. All statistical analysis was performed in Prism for Windows, version 9. The numerical value of the p-value is either used directly or a star signifies a p-value <0.05 that was considered as statistically significant. The number of replicates in the different assays may vary since not all animals were used in all assays.

## 3 Results

### 3.1 Organ distribution of PM

The size distributions of the used SPM are presented in Suppl. Fig. S2. Since particle mass is proportional to the diameter cubed, when exposing animals to the same mass concentration, they are exposed to more individual particles in the nano-sized SPM exposure groups. The measured values of the mean SPM mass concentration in the exposure chamber were: 248 ± 66 μg/m^3^ for nano-fluorescent SPM, 270 ± 65 μg/m^3^ for micro-fluorescent SPM, 209 ± 37 μg/m^3^ for nano-magnetic SPM and 221 ± 27 μg/m^3^ for micro-magnetic SPM.

**Fig. 1:**
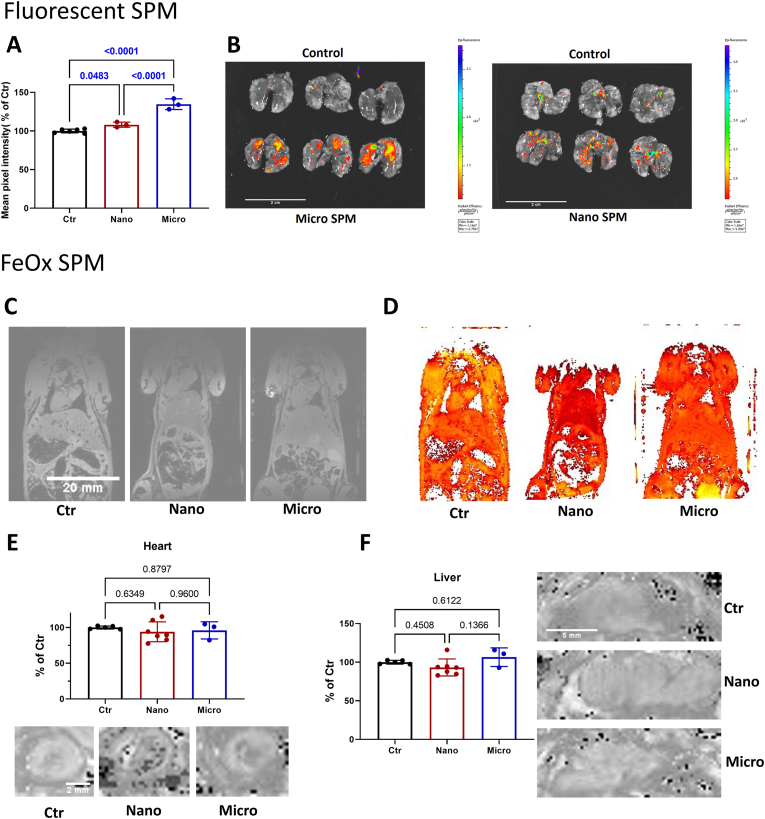
**Distribution of particles with different size after inhalation in the mouse body.** Lungs of fluorescent nano- and micro-SPM exposed animals were subjected to fluorescence imaging (B), and the mean pixel intensity was recorded (A). Mice exposed to magnetic nano- and micro-SPM underwent magnetic resonance imaging (MRI) of the abdomen measuring T_2_∗-relaxation maps. Representative T_2_∗-weighted images (C) with the corresponding R_2_∗ parameter maps (D). Images of the heart (E) and liver (F) sections together with quantifications zoomed in from C/D showing pixels with threshold contrast. The scale bar for B is 2 cm, for C and D is the same at 20 mm, for E is 2 mm and for F is 5 mm. Data are presented as mean ± SEM from n = 3–7 animals per group. P values for individual comparisons are shown indicating statistical significance obtained by one-way ANOVA with Tukey's multiple comparison analysis.

After the exposure to fluorescent micro- or nano-SPM, mice were sacrificed and extracted lungs were imaged with a fluorescence imager (Fig. 1A and B). The lungs of mice exposed to micro-SPM showed a pronounced difference in fluorescence when compared to non-exposed mice, while the lungs of nano-SPM-exposed mice showed a more subtle increase in fluorescence intensity (Fig. 1A). The more pronounced increase in fluorescence intensity after the micro-SPM exposure points to the accumulation of the particles in the lung, while the lower fluorescence after nano-SPM exposure points to the migration of particles from the lung tissue into circulation.

After the exposure to magnetic micro- or nano-SPM, mice were subjected to a whole-body rodent MRI depicting the abdomen of the mice (Fig. 1C and D). The T_2_∗ relaxation values of the tissue were measured, enabling the calculation of R_2_∗ parameter maps. We hypothesized that the iron particles may accumulate in the heart (Fig. 1E) and liver (Fig. 1F) when they enter the body, and therefore, these organs were examined for contrast changes due to the presence of magnetic SPM. No statistically significant changes were observed in either organ, although a trend toward a reduction in R_2_∗ values was observed in the nano-SPM-exposed mice.

### 3.2 Vascular function in nano- and micro-SPM exposed mice

**Fig. 2:**
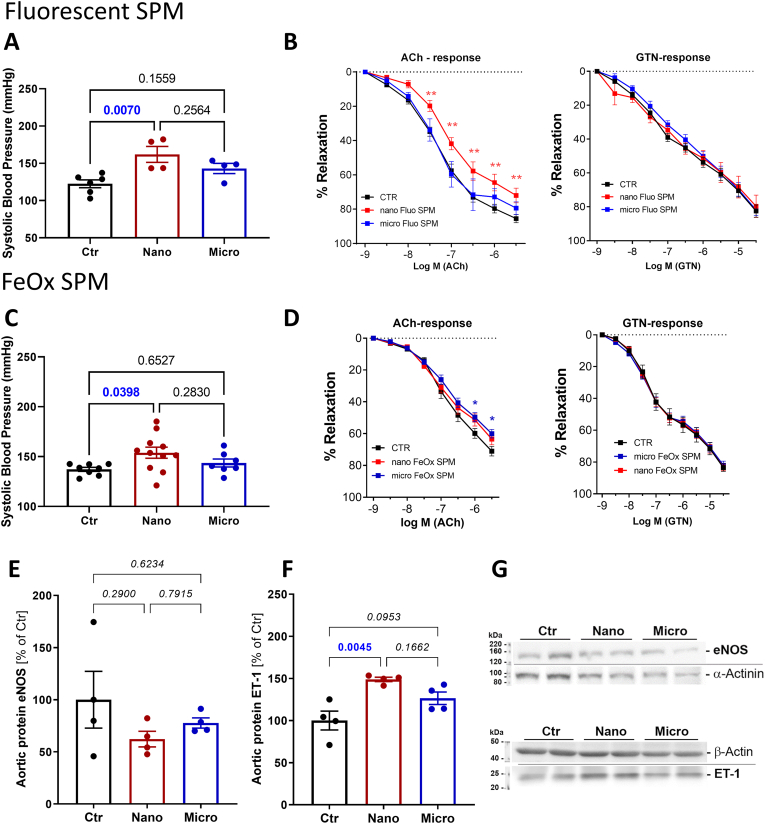
**Effects of SPM different size on vascular function.** Systolic blood pressure was measured in mice exposed to fluorescent SPM (A) and magnetic SPM (C). Vascular function was measured in isolated aortic rings of fluorescent SPM (B) and magnetic SPM (D). The endothelium-dependent relaxation in the presence of acetylcholine (ACh) and the endothelium-independent relaxation in the presence of nitroglycerin (GTN) are shown for both type of SPM exposure. Western blot quantifications for magnetic SPM-exposed mice aortic endothelial nitric oxide synthase (eNOS) (E) and endothelin 1 (ET-1) (F) expression levels are shown together with the representative blots (G). Data are presented as mean ± SEM from aortic rings of n = 4–9 mice per group (B, D), or the mouse number is shown by jitter plots for other parameters (n = 4–11 mice per group). P values for individual comparisons are shown, indicating statistical significance, or asterisks are used: ∗ (p < 0.05), ∗∗ (p < 0.01) obtained by one-way ANOVA with Tukey's multiple comparison analysis for A, C, E and F, and by two-way ANOVA with Tukey's multiple comparisons test for B and D.

Systolic blood pressure, as measured by the tail-cuff method, was increased after exposure to both the fluorescent and magnetic nano-sized SPM (Fig. 2A and C). Blood pressure was not changed after exposure to fluorescent and magnetic micro-sized SPM. Endothelium-dependent vascular relaxation achieved through ACh titration showed a right shift after exposure to fluorescent nano-SPM but not after the exposure to fluorescent micro-SPM (Fig. 2B). The endothelium-independent vascular relaxation in response to nitroglycerin was not changed upon fluorescent SPM exposure. The exposure to magnetic nano- and micro-SPM caused a right shift in the endothelium-dependent relaxation curve by trend, but no clear pattern emerged (Fig. 2D). The exposure to magnetic SPM also did not affect the endothelium-independent vascular relaxation in response to nitroglycerin. Aortic protein expression of endothelin 1 (ET-1) was also increased in mice exposed to the magnetic nano-SPM, together with a decrease in endothelial nitric oxide synthase (eNOS) by trend (Fig. 2E and F).

### 3.3 Higher ROS formation in different tissues of PM exposed mice

**Fig. 3:**
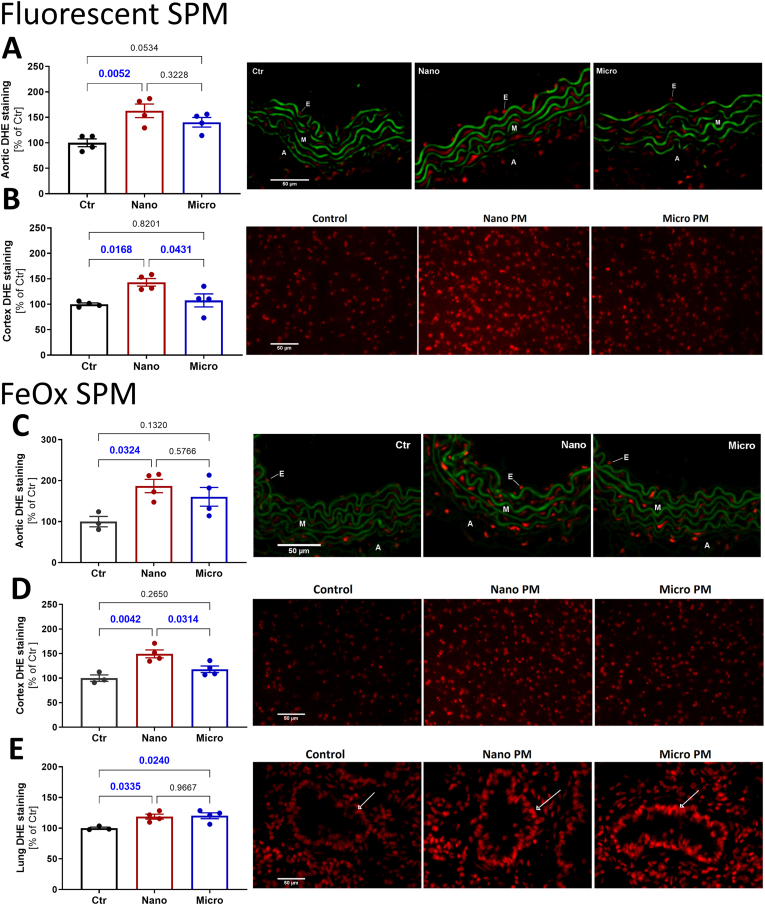
**Effects of fluorescent SPM of different sizes on aortic, cortical and pulmonary oxidative stress.** Dihydroethidium (DHE) fluorescence microtopography was used to assess the oxidative stress burden in different tissues. Quantification of oxidized DHE fluorescence in aortic (A) and cortical (B) tissue of fluorescent SPM exposed animals and in aortic (C), cortical (D), and pulmonary (E) tissue of magnetic SPM-exposed animals are shown together with representative image. Green color in panels A and C reflects the autofluorescence of the basal laminae. The arrows in panel E indicate the bronchioles. The scale bar for all images is the same at 50 μm. Data are presented as mean ± SEM and the mouse number is shown by jitter plots (n = 3–4 mice per group). P values for individual comparisons are shown indicating statistical significance obtained by one-way ANOVA with Tukey's multiple comparison analysis.

Dihydroethidium (DHE) staining was used to assess spatial ROS levels in aortic, pulmonary, and cortical tissue of SPM-exposed mice. Fluorescent nano-SPM showed a significant increase in oxidized DHE-derived fluorescence in both aortic and cortical tissue (Fig. 3A and B). The fluorescent micro-SPM did not change the ROS levels in these tissues compared to the non-exposed control. Magnetic nano-SPM exposure caused again an increased oxidized DHE fluorescence signal (Fig. 3C and D), which was absent upon exposure to magnetic micro-SPM. In the pulmonary tissue, the magnetic micro- and nano-SPM showed an increase in oxidized DHE-derived fluorescence (Fig. 3E). The lung tissue of fluorescent SPM-exposed animals could not be evaluated due to excessive fluorescence background originating from the accumulation of fluorescent particles in the lungs of exposed mice.

### 3.4 Protein markers of enhanced ROS formation and inflammation in cardiac and pulmonary tissue

**Fig. 4:**
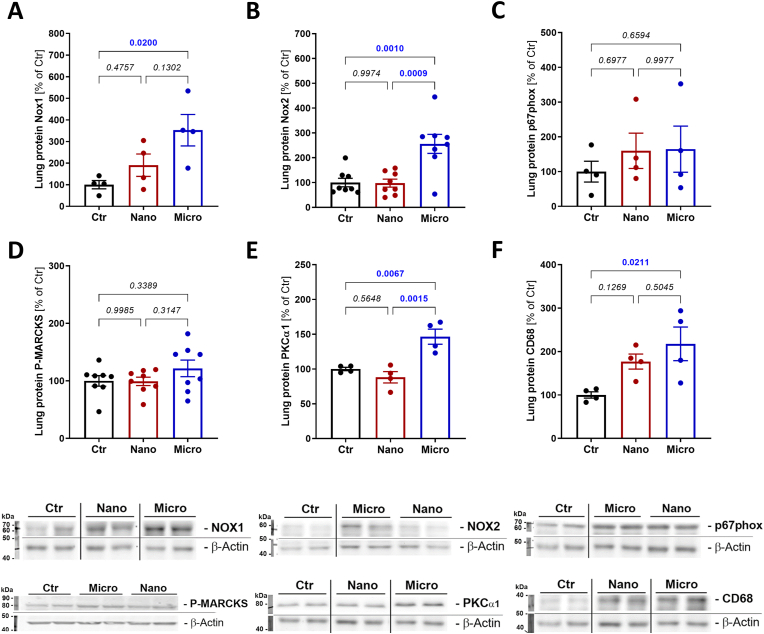
**Effects of magnetic SPM with different sizes on pulmonary protein expression.** Western blot analysis of the pulmonary NADPH oxidase subunits NOX1 (A), NOX2 (B) and p67phox (C), phosphorylated myristoylated alanine-rich C-kinase substrate (P-MARCKS) (D), protein kinase C alpha1 (PKCα1) (E), and cluster of differentiation 68 (CD68) (F) are shown for the magnetic SPM exposed mice. Data are presented as mean ± SEM, and the mouse number is shown by jitter plots (n = 4–8 mice per group). P values for individual comparisons are shown, indicating statistical significance obtained by one-way ANOVA with Tukey's multiple comparison analysis.

NADPH oxidase subunits NOX1 and NOX2 protein expression was elevated in the lung tissue of magnetic micro-SPM, but not in the lung tissue of nano-SPM exposed mice (Fig. 4A and B). In addition, the NADPH oxidase subunit p67phox was measured but did not show a trend in either micro- or nano-SPM exposure groups (Fig. 4C). Protein kinase C alpha 1 (PKCα1), which promotes NADPH oxidase complex formation, was also elevated in the lung tissue but phosphorylated myristoylated alanine-rich C-kinase substrate (P-MARCKS), a marker of PKCα1 activity, did not change (Fig. 4D and E). CD68 was also elevated in the lungs of micro-SPM-exposed mice, indicating local inflammation initiation (Fig. 4F).

**Fig. 5:**
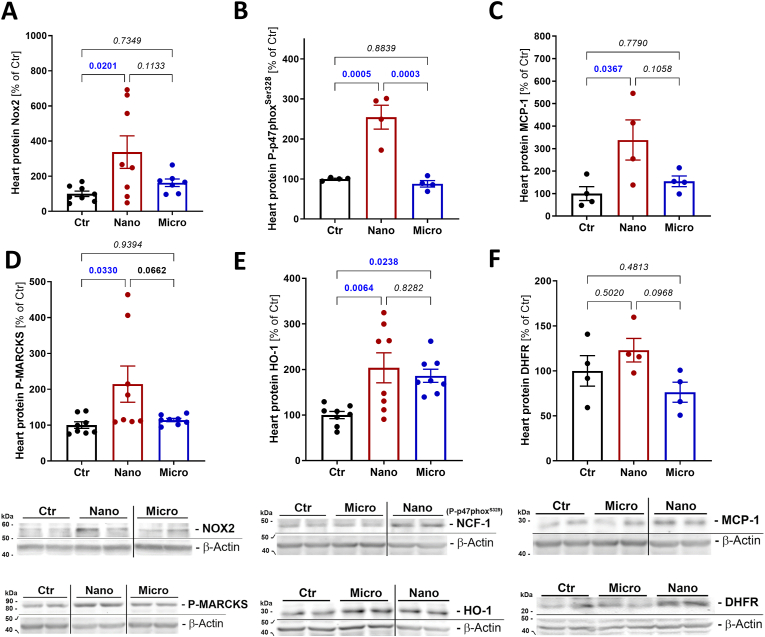
**Effects of magnetic SPM with different sizes on cardiac protein expression.** Western blot analysis of the pulmonary NADPH oxidase subunits NOX2 (A) and phosphorylated p47^phox^ (NCF-1) (B), monocyte chemoattractant protein-1 (MCP-1) (C), phosphorylated myristoylated alanine-rich C-kinase substrate (P-MARCKS) (D), heme oxygenase-1 (HO-1) (E), and dihydrofolate reductase (DHFR) (F) are shown for the magnetic SPM-exposed mice. Data are presented as mean ± SEM, and the mouse number is shown by jitter plots (n = 4–8 mice per group). P values for individual comparisons are shown, indicating statistical significance obtained by one-way ANOVA with Tukey's multiple comparison analysis.

In cardiac tissue, it was only the nano-SPM that produced a significant effect. NADPH oxidase subunit NOX2 and the phosphorylated p47^phox^ at serine 328 showed a significant increase in protein expression (Fig. 5A and B), pointing to the activation of the ROS-producing complex. Monocyte chemoattractant protein-1 (MCP-1), a marker of inflammation, was also elevated in the cardiac tissue of nano-SPM, but not in the micro-SPM exposed mice (Fig. 5C). P-MARCKS' expression was increased upon nano-SPM exposure, indicating kinase activity (Fig. 5D). Heme oxygenase 1 (HO-1) was significantly upregulated in the hearts of both nano- and micro-SPM-exposed mice, indicating the activation of the antioxidant defense through the Nrf2 pathway (Fig. 5E). The expression of dihydrofolate reductase (DHFR), was not observed to be significantly changed (Fig. 5F).

### 3.5 CVD incidence from UFP

**Fig. 6:**
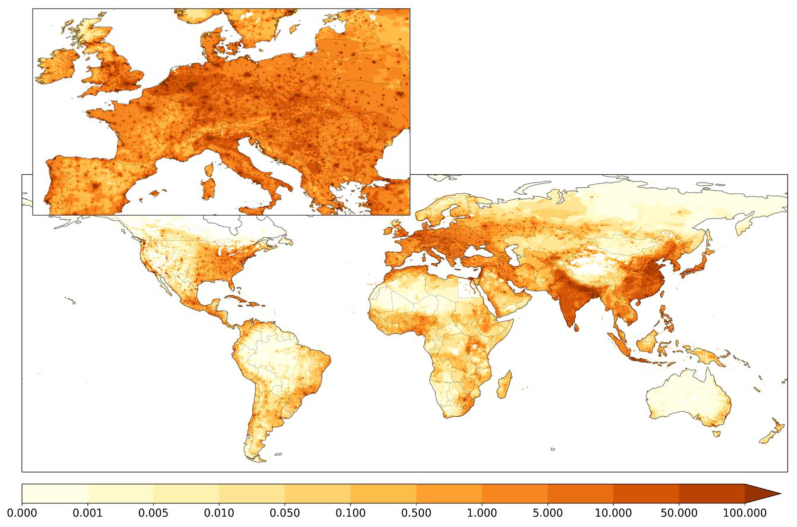
**European and global, annual CVD incidence attributed to UFP.** Units are the number of cases per surface area of 10 km × 10 km. These results suggest that UFP exposure and CVD impacts are predominant in the urban environment.

We combined the downscaled UFP exposure data (at 0.1° global resolution) with hazard ratios of the increased risk for incident CVD adopted from an epidemiological cohort study [51] and computed the attributable fractions as a function of UFP number concentrations. Results are shown in Fig. 6, indicating that CVD incidence from UFP exposure is primarily a consequence of air pollution in urban centers. The cohort study was performed in cities in the Netherlands, and considering it is the only one of its kind, we assume it is representative of the population in Europe. For the 27 countries of the European Union (EU-27), we estimate a UFP-attributable CVD incidence of 419 (95 % CI: 78–712) thousand per year for a total population of 446 million. The highest incidence occurs in Germany, with 82 (15–142) thousand per year, followed by Italy with 67 (13–115) and France with 42 (8–71) thousand per year. Since these are relatively populous countries in the EU-27, we additionally estimated the per capita incidence. We found that it is highest in Greece, with 171 (37–256) per 100,000 population per year, followed by Hungary, with 165 (34–253) and Bulgaria with 159 (30–267) per 100,000 annually, whereas it is lowest in Ireland with 33 (7–53) and Finland with 43 (8–74) per 100,000 population per year. If the Dutch epidemiological study would also be representative worldwide (absent dedicated cohort studies), we derive a yearly global CVD incidence of 5.6 (95 % CI: 1.1–9.3) million, attributable to the exposure to UFPs (Fig. 6). Since the global CVD incidence from all causes is 47.1 (95 % UI: 40.9–53.9) million per year [52], the UFP-attributable incidence amounts to a fraction of about 11–12 % of the total. For data at the country level, we refer to Suppl. Fig. S3 and extended results in the **online supplement**.

## 4 Discussion

### 4.1 Health implications

In this work, we investigated the biological toxicity of nano-versus micro-sized synthetic particles, emphasizing their potential to transmigrate through the lung epithelium into the bloodstream and cause damage to other, remote organs (e.g. the aorta, heart, and brain). We used fluorescence-labeled or magnetic nano- and micro-sized particles to trace their biodistribution. We found support for the transmigration of nano-sized fluorescent SPM in contrast with micro-sized particles. This was indicated e.g. by a lower fluorescence signal in the lung, but a more pronounced increase in blood pressure and endothelial dysfunction, and detrimental effects through higher ROS levels in aortic and cortex tissues, also reflected by higher expression of ROS-producing enzymes. Likewise, magnetic nano-sized SPM showed a tendency to accumulate in the liver and heart compared to the micro-sized SPM, with higher blood pressure, higher aortic and cerebral ROS levels, and dysregulated cardiac protein expression. These data indicate that nano-sized SPM penetrates the respiratory epithelium, transmigrates into circulation, and affects distant organs, whereas micro-sized SPM primarily affects the lungs. Further, we performed a preliminary translational study indicating that the UFP fraction of PM is a major contributor to CVD incidence in Europe and the world. The translational aspect of these findings remains to be fully established, noting that there is a particular need for longitudinal studies.

The research on air pollution, especially particulate matter (PM), has gained attention from the medical and public health research communities due to its association with morbidity and excess mortality, marking it as a global risk factor comparable to tobacco smoking [52]. There is substantial epidemiological and clinical evidence supporting the significant contribution of PM to adverse human health effects, impacting both the respiratory and remote organ systems [53]. Numerous studies examined the differential effects of PM composition, providing an overview of the role and contribution of various toxicants carried by airborne PM [[54], [55], [56]]. Conversely, the studies addressing the effects of differential PM size have thus far been inconclusive. A possible reason is that air pollution-derived UFPs exhibit a high load-to-mass ratio of surface toxicants interacting with biological tissue, surpassing size effects [12]. Further, the use of biologically inert PM, e.g. for contrast agents, drug delivery systems and food or cosmetic stabilizers has generated contradicting findings [57,58]. Especially in preclinical research, chemically inert particles, such as TiO_2_, silver, gold, or synthetic carbon/plastic particles have been used [[59], [60], [61]].

Here, we provide novel insights into the direct effects of PM size on the presence of elevated ROS levels and inflammation in the lung and remote organ systems such as the aorta, heart, and brain. DHE staining provides only an overview of the cumulative H_2_O_2_ and O_2_·^-^ production, and is not representative of the cellular redox state for which the measurement of ROS formation and degradation by antioxidants should be assessed [62]. Using synthetic particles, i.e. apart from the hazardous chemical components present in air pollution, we show that the size of PM plays a significant role in detrimental effects on multiple organ systems. This may indicate additive or synergistic adverse effects by ultrafine or nano-particles with elevated toxicant loads due to a high surface/mass ratio associated with high particle number concentrations [33,63]. Ambient particles contain chemically active substances (such as transition and heavy metals, peroxides, quinones, endotoxins) known to additionally induce inflammation and promote oxidative stress conditions [[64], [65], [66], [67], [68]]. This has direct consequences for exposure-health associations as shown by more frequent cardiorespiratory emergency department visits of exposed individuals in dependence of a higher oxidative potential of PM_2.5_ [69]. In general, the oxidative potential of PM_2.5_ and UFPs contributes to the development of chronic diseases but also causes acute cardiovascular events [70,71]. Oxidative potential of ambient air pollution particles shows large regional and seasonal variations [72,73]. Of note, in the present study we exclude the contribution of the particle oxidative potential to the observed functional and biochemical changes since the particles used were of synthetic origin and chemically inert.

### 4.2 UFP pathomechanisms

Oxidative stress and inflammation play a major role in vascular dysfunction as they interfere with the pathways regulating vascular tone. Nitric oxide (·NO), as an important signaling molecule, is susceptible to oxidative stress, as the reaction with superoxide (O_2_·^-^) not only creates peroxynitrite (ONOO^−^) at the expense of ·NO, but it also impairs ·NO production by uncoupling eNOS [9,74]. In the present study, the origin of O_2_·^-^ can be attributed to the activation of the NADPH oxidase (NOX1/2), supported by the elevated 10.13039/100012258PKC activity through phosphorylation of MARCKS and p47^phox^ at serine 328, although the exact cellular origin cannot be assigned since tissue homogenates were used. The enhanced levels of ROS in the aortic tissue observed after exposure to nano-sized SPM correlate with the impairment of endothelium-dependent vasodilation and the lowering of eNOS expression by trend. The upregulation of DHFR by trend in the heart tissue of mice exposed to nano-sized SPM may indicate a counter-regulatory attempt of the cell to compensate for the impaired endothelial function by “recycling” dihydrobiopterin (BH_2_) back to tetrahydrobiopterin (BH_4_) for proper eNOS function [75]. Overall, the enhanced oxidative stress condition is reflected by the upregulation of the stress response and antioxidant enzyme HO-1 by nano-sized SPM in the heart.

Elevation of ET-1 expression, a potent vasoconstrictor that is upregulated by oxidative stress [74], points to the ability of nano-sized PM to promote endothelial cell activation via NOX-derived ROS [76] and oxidative eNOS uncoupling by S-glutathionylation [77] contributing to vascular dysfunction. ET-1 is also a mitogen [78], and some studies have shown that PM exposure can induce vascular hypertrophy [79]. In general, PM-induced inflammation also promotes vascular dysfunction [[80], [81], [82]], e.g. by an oxidative burst of activated leukocytes upon tight adhesion to the endothelium or infiltration into the vascular wall [83]. Elevated levels of CD68 in the pulmonary tissue of micro-sized SPM point to local inflammation in the lung, which is also accompanied by an increase in ROS production, while increased expression of MCP-1 in the cardiac tissue of nano-sized SPM points to the ability of ultrafine particles to transmigrate through the lung epithelium, reach the circulation and cause inflammation and remote organ damage, notably in the cardiovascular system. Elevation of CD68 was also found in the brain of PM_2.5_-exposed Alzheimer's disease prone mice together with other cytokines and chemokines [84]. Higher MCP-1 levels were found in rats exposed to concentrated ambient fine particulates accompanied by elevated TNF-α and diminished IL-10 levels [85]. Likewise, MCP-1 induction was observed in cultured macrophages exposed to transition-metal rich residual oil fly ash particles together with TNF-α, IL-6 and IL-1β levels [86]. CD68 is highly expressed in monocytes and phagocytes and, therefore, indicative of enhanced infiltration of these immune cells into tissues. MCP-1 is one of the key chemokines that regulate the migration and infiltration of monocytes/macrophages. These markers also support the concept that inhaled particles are taken-up by resident macrophages in the lung [87], which then cross the air-blood barrier, reach the circulation and remote organs and most likely end-up in the liver [88].

In addition to inflammatory and NOX-mediated damage, nano- (to a certain extent also micro-) particles alter redox homeostasis, cause mitochondrial oxidative stress and damage and thereby dysfunction of mitochondrial processes [[89], [90], [91]]. Nano-particles exacerbate ischemia/reperfusion damage in isolated heart tissue by changes in the opening probability of the mitochondrial permeability transition pore [92]. Mitochondria were previously shown to be a prominent source of ROS in different PM exposure models [93]. When human bronchial epithelial cells were repeatedly exposed to low concentrations of fine (2.5–0.18 μm) or quasi-ultrafine particles (0.18 μm) no significant cytotoxicity, apoptosis or changes of mitochondrial membrane potential (ΔΨm) and intracellular ATP content were observed [91]. However, oxidative phosphorylation, mitochondrial mass and mitochondrial superoxide anion generation were increased, also leading to altered mitochondrial dynamics and NRF2-dependent stress response. However, another study found that exposure to residual oil fly ash impairs cardiac mitochondrial function by decreasing respiration and ATP synthesis, all of which caused altered response to the pacing drug isoproterenol and changes in contractile reserve [94].

Another pathway by which PM can influence the cardiovascular system is by activation of the sympathetic nervous system (SNS) and the amygdala stemming from the translocation of the UFPM through the olfactory nerve [95]. The activation of the SNS leads to the release of catecholamines, causing vascular constriction, enhanced blood pressure and vascular inflammation [96,97], as observed here, together with the increase in cortical ROS production. In addition, modulation of the SNS can disrupt the circadian rhythm, further disrupting cardiovascular redox balance by phase shifts of genes encoding for ROS-producing and degrading proteins [98]. Likewise, UFPM can cross the blood-brain barrier [99] and vehicle exhaust particles can alter the permeability of the blood-brain barrier [100] to cause cerebral oxidative stress and neuroinflammation [101]. Air pollutants cause circadian rhythm impairment by adverse redox regulation of the clock core components such as period, cryptochrome, clock and BMAL1. It was also previously observed that SPM can disrupt cardiac function through myocardial injury and apoptosis via ROS [102].

In support of our suggested pathomechanisms, previous work showed that nanometer-sized PM (<200 nm) from urban traffic causes oxidative stress through enhanced 4-hydroxynonenal and 3-nitrotyrosine levels, and inflammation in olfactory epithelial cells with subsequent activation of astrocytes, microglia and upregulation of markers of neuroinflammation [103]. Inhalation of airborne, iron-rich nanoparticles (15–40 nm) led to mitochondrial accumulation in the hearts of exposed healthy subjects and an increase of markers of cardiac oxidative stress and cardiovascular disease [104]. A study found that small gold nano-particles accumulate in atherosclerotic plaque areas of ApoE knockout mice on a high-fat diet (instillation of the gold particles) and in surgical specimens of carotid artery disease from patients at risk of stroke [32]. In contrast, for larger nanoparticles it was reported that exposure of healthy subjects to small and ultrasmall graphene oxide nanosheets (thickness 1.2–1.6 nm, lateral size 427 vs 153 nm) did not affect heart rate, blood pressure, lung function and inflammatory markers, and only minor changes of the plasma proteome and *ex vivo* thrombus formation were observed [105]. The reactive oxygen species and oxidative damage resulting from different PM categories in the lung lining fluid can be predicted using computational models supporting the formation of superoxide, hydrogen peroxide, hydroxyl radicals and 3-nitrotyrosine in this model [106,107]. The model even predicted the severity of chronic lung diseases by considering endogenous inflammatory processes in regionally different populations with varying PM exposure concentrations [68]. The most prominent human health effects of 10 engineered nanomaterials were previously summarized by a multi-laboratory toxicological assessment using *in vivo* and *in vitro* approaches [108].

### 4.3 Biomedical impact of particle size

Research during the last two decades revealed the difference in toxicity between PM_10_ and PM_2.5_, as the larger particles do not penetrate as deeply into the respiratory tract and are more easily eliminated [[109], [110], [111]]. It is important to recall that the larger size fraction of PM_10_ (which encompasses PM_2.5_) presents a significant risk factor for many diseases [112], also due to the loading with environmental toxins. The health differences between UFPs and PM_2.5_ remain understudied, and only a few cohort studies have been reported. This is mainly because UFPs were, until recently, not routinely measured in air pollution networks, and accordingly, are not part of the legislature, and their health risk may be strongly underestimated. A study from China showed that only particles smaller than 1 μm (PM_1_) are positively associated with cardiovascular morbidity [113]. Another study from China demonstrated a positive association between PM in the size range of 0.25–0.5 μm and cardiovascular mortality, while the mortality from respiratory diseases was insignificant [114]. A study in Erfurt, Germany, found a similarly higher risk for cardiovascular mortality from UFPs than respiratory mortality [115]. The results of these two studies support the transmigration of nano-sized particles through the lung and direct damage to remote organs.

A recent study in Copenhagen, Denmark, established that hospital admissions for cardiovascular and respiratory diseases positively correlated with increased UFP concentrations [116]. Interestingly, after adjustment for PM_2.5_, all associations with respiratory diseases decreased. In contrast, associations with cardiovascular disease increased, pointing to a direct effect of UFPs, but not of larger PM, on the cardiovascular system. Data from a Dutch cohort corroborate the findings of cardiovascular disease association with UFPs rather than coarser PM, highlighting this through a two-pollutant model where UFPs remained the only positively associated variable for cardiovascular risk [51]. On the other hand, in a cohort from Toronto, Canada, no change in the association between UFPs and acute myocardial infarction and congestive heart failure was observed after correcting for exposure to PM_2.5_ and ^•^NO_2_ [117], while a recent study that size-separated micro-from nano-particles corroborated the association of UFPs with increased mortality risk in Canada's two largest cities [118]. The European Study of Cohorts for Air Pollution Effects (ESCAPE) did not find a correlation between PM_2.5_ concentrations and all cardiovascular disease deaths [119].

### 4.4 CVD incidence from UFP exposure

Our European and global calculations of UFP exposure and potential consequences for CVD incidence are presented in the Supplement. These results are associated with considerable uncertainty and are intended to derive a first-order estimate of cardiovascular disease outcomes in humans. The annual average UFP concentration in the cohort study of Downward et al. (2018) [51], performed among residents of major metropolitan areas in the Netherlands, was 11,110 (±2400) particles cm^−3^. This is at the higher end of that observed in European cities (e.g., between Milan and Barcelona) but lower than in Chinese cities and Arabian Gulf states, for example [120,121]. It captures a good part but not the full spread we find in global, annual UFP exposure, and clearly, additional cohort studies are needed to increase statistical robustness. Even though the Downward et al. (2018) [51] study is the only one available that directly attributes CVD incidence to UFP, it should be noted that these outcomes are qualitatively consistent with epidemiological studies that relate UFP to hypertension and diabetes [122,123] and congestive heart failure and acute myocardial infarction [124], as well as enhanced mortality risk [125]. Hence, the quantitative outcomes presented here may be uncertain, but they corroborate the high likelihood that exposure to UFP contributes to CVD incidence.

### 4.5 Limitations of the study

The nano-SPM (mean diameter of 200–250 nm) used for the present study are not “ultrafine” particles by definition since this term is generally used for particles ≤100 nm. However, the particle size distribution admitted in our experiments included UFPs even though the mass mean size was larger. The choice of a chemically inert synthetic material was based on the primary objective of the present study to determine size effects instead of additional mechanisms through the chemical loading of particles. Previous work reports a smaller mean diameter for efficient transmigration of inhaled nanoparticles of around 50 nm [32], although a systematic review on the deposition of particles in different lung tracts reported a range of up to 200 nm to reach the alveolar region and transmigrate into the bloodstream [126]. Our study encountered several limitations related to PM exposure. A major challenge was achieving precise exposure concentrations due to the non-uniform size distribution of PM, as larger particles disproportionately contribute to the measured mass concentrations, while smaller particles dominate the number distributions. This complicates analyses based on average PM diameters. The low PM amount available through inhalation was insufficient for clear MRI contrast, with mouse exposure levels far below human application thresholds of contrast agents by intravenous administration. This limitation could be addressed by longer exposure times, though uncertainties remain due to poorly characterized clearance processes. The lack of pronounced fluorescence signals in mouse lungs might be due to relatively high exhalation rates of nanoparticles, rather than general transmigration into the bloodstream. Whereas endotoxin contamination of the synthetic particle preparations can be excluded (this was highly purified material as advertised on the companies’ webpages), the different coating of the iron oxide particles (silica-versus polystyrene-encapsulation) must be mentioned and could have impacted the observed effects. Further, we cannot exclude that appreciable numbers of nano-SPM reached the circulation via oral uptake, e.g. from fur grooming, since we used a whole body exposure system (which would still not explain the lack of fluorescence in the lung upon exposure to nano-SPM). Oral uptake would then allow the particles to reach the circulation by penetration of the gut, which has a higher permeability than the lung epithelium. The changes in oxidative stress and inflammation markers in the pulmonary and cardiac tissue are derived from the whole tissue homogenate and do not represent the expression of only one cell type. Finally, some parameters are underpowered (e.g. n = 3–4), which may result in reduced significance. Despite these uncertainties, our findings of nano-sized SPM effects on blood pressure and oxidative stress in remote organs directly support their efficient transmigration, aligning with human data linking UFP exposure to cardiovascular risks. A detailed consideration of all limitations can be found in the **online supplement**.

### 4.6 Conclusions and clinical implications

Our study highlights the distinct biological impacts of nano- and micro-sized synthetic particles in exposed mice, suggesting significant human health effects. Nano-sized particles can transmigrate through lung epithelium into the bloodstream, affecting distant organs such as the aorta, heart, and brain. This suggests significant direct systemic impacts, extending beyond the pulmonary effects typically associated with larger micro-sized particles, which tend to directly affect the respiratory system, causing pulmonary damage with localized and possible indirect systemic health outcomes.

Our data show that nano-particles (in our study with a mean mass-related diameter <250 nm) enhance the oxidative stress and inflammatory parameters in remote organ systems, while micro-particles (in our study with mean mass-related diameters of 2.1 and 4.1 μm) primarily impact the pulmonary system. The distinction is important in defining future studies, as different organ systems could be impacted by PM of varying sizes through different mechanisms, also leading to a differential increase in the risk of specific disease categories, notably cardiovascular versus respiratory diseases, as supported by several clinical/epidemiological studies on UFP exposure–health associations. Our preliminary health burden estimate suggests significant impacts of ultrafine (nano-)particles on cardiovascular disease incidence at the European and global scales.

Our results are unexpected since, thus far, direct functional and biochemical effects of chemically inert particles were assigned to diameters below 100 nm. Importantly, previous work has shown that chemically inert gold particles with a diameter of 5 nm show significantly more pronounced uptake into the circulation by higher levels in blood and urine in exposed healthy individuals than particles with a size of 30 nm [32]. Nevertheless, also nano-particles ≥100 nm may transmigrate efficiently, although the deposited particles tend to concentrate in the upper pulmonary areas such as the tracheobronchial airway and to a lesser extent in the alveolar region [127].

Clinically, these findings emphasize the need for healthcare frameworks to consider particle size in risk assessments and, ultimately, in air quality directives. The ability of nano-sized particles to cause systemic harm underscores their role in causing and/or exacerbating cardiovascular conditions and necessitates targeted public health strategies to mitigate their effects. This warrants the comprehensive incorporation of UFP measurements into air quality monitoring stations, enabling the development of enhanced spatial and temporal resolution exposure distributions to study long-term associations in epidemiological cohort studies. However, this may pose challenges in many low- and middle-income countries, where PM_2.5_ monitoring is frequently not implemented. Our modeling approach to address exposure-health interactions may help fill these gaps.

## CRediT authorship contribution statement

**Marin Kuntic:** Writing – review & editing, Writing – original draft, Supervision, Investigation, Formal analysis, Data curation, Conceptualization. **Ivana Kuntic:** Writing – original draft, Investigation, Data curation. **Dirk Cleppien:** Writing – original draft, Formal analysis, Data curation. **Andrea Pozzer:** Writing – original draft, Formal analysis, Data curation, Conceptualization. **David Nußbaum:** Methodology, Investigation, Data curation. **Matthias Oelze:** Writing – review & editing, Methodology, Investigation, Formal analysis, Data curation. **Tristan Junglas:** Methodology, Investigation, Data curation. **Lea Strohm:** Methodology, Investigation, Data curation. **Henning Ubbens:** Methodology, Investigation, Data curation. **Steffen Daub:** Writing – review & editing, Supervision, Funding acquisition. **Maria Teresa Bayo Jimenez:** Methodology, Investigation, Data curation. **Sven Danckwardt:** Writing – review & editing, Methodology, Funding acquisition. **Thomas Berkemeier:** Writing – review & editing, Writing – original draft. **Omar Hahad:** Writing – review & editing, Funding acquisition. **Matthias Kohl:** Writing – original draft, Methodology, Investigation, Formal analysis, Data curation. **Sebastian Steven:** Writing – review & editing, Methodology, Funding acquisition. **Albrecht Stroh:** Writing – review & editing, Writing – original draft, Funding acquisition, Formal analysis, Conceptualization. **Jos Lelieveld:** Writing – review & editing, Writing – original draft, Supervision, Funding acquisition, Formal analysis, Conceptualization. **Thomas Münzel:** Writing – review & editing, Writing – original draft, Supervision, Funding acquisition, Formal analysis. **Andreas Daiber:** Writing – review & editing, Writing – original draft, Supervision, Project administration, Methodology, Funding acquisition, Formal analysis, Conceptualization.

## Funding

A.D. and T.M. were supported by vascular biology research grants from the 10.13039/501100008454Boehringer Ingelheim Foundation for the collaborative research group “Novel and neglected cardiovascular risk factors: molecular mechanisms and therapeutics” and through continuous research support from 10.13039/100017578Heart Foundation Mainz (mostly dedicated to the purchase of the exposure system and to the salary of M.K.). I.K. and M.T.B.J. hold/held TransMed PhD stipends funded by the 10.13039/501100008454Boehringer Ingelheim Foundation. L.S. and H.U. hold TransMed PhD stipends funded by the Else-Kröner-Fresenius Foundation. S.St. holds an excellence stipend of the Else-Kröner-Fresenius Foundation (2021_EKES.04). T.J. holds a MD stipend funded by the Heart Foundation Mainz. T.M. is PI and A.D. and M.K., O.H. and S.Daub are (Young) Scientists of the 10.13039/100010447DZHK (German Center for Cardiovascular Research), Partner Site Rhine-Main, Mainz, Germany. A.P., T.B., J.L., T.M. and A.D. were funded by the environmental research consortium MARKOPOLO under the HORIZON call HLTH-2024-ENVHLTH-02-06 (Grant Agreement Number 101156161) funded by the 10.13039/501100000780European Union and the Swiss 10.13039/501100007352State Secretariat for Education, Research and Innovation (SERI). Large instrument funding by the 10.13039/501100001659German Research Foundation (10.13039/501100001659DFG, project 251892550 to S. Danckwardt). Work in the lab of S.Danckwardt is supported by the EU 10.13039/501100007601Horizon 2020 Innovative training network ‘Thromboinflammation in Cardiovascular disorders’ (TICARDIO, Marie Skłodowska-Curie grant agreement No 813409). The work was also supported by the 10.13039/501100000921COST Action CA20121 – BenBedPhar and the environmental network EXPOHEALTH funded by the Science Initiative of the state Rhineland-Palatinate, Germany.

## Declaration of competing interest

The authors declare the following financial interests/personal relationships which may be considered as potential competing interests:A.D. and T.M. were supported by vascular biology research grants from the 10.13039/501100008454Boehringer Ingelheim Foundation for the collaborative research group “Novel and neglected cardiovascular risk factors: molecular mechanisms and therapeutics” and through continuous research support from 10.13039/100017578Heart Foundation Mainz (mostly dedicated to the purchase of the exposure system and to the salary of M.K.). I.K. and M.T.B.J. hold/held TransMed PhD stipends funded by the 10.13039/501100008454Boehringer Ingelheim Foundation. L.S. and H.U. hold TransMed PhD stipends funded by the Else-Kröner-Fresenius Foundation. S.St. holds an excellence stipend of the Else-Kröner-Fresenius Foundation (2021_EKES.04). T.J. holds a MD stipend funded by the Heart Foundation Mainz. T.M. is PI and A.D. and M.K., O.H. and S.Daub are (Young) Scientists of the 10.13039/100010447DZHK (German Center for Cardiovascular Research), Partner Site Rhine-Main, Mainz, Germany. A.P., T.B., J.L., T.M. and A.D. were funded by the environmental research consortium MARKOPOLO under the HORIZON call HLTH-2024-ENVHLTH-02-06 (Grant Agreement Number 101156161) funded by the 10.13039/501100000780European Union and the Swiss 10.13039/501100007352State Secretariat for Education, Research and Innovation (SERI). Large instrument funding by the 10.13039/501100001659German Research Foundation (10.13039/501100001659DFG, project 251892550 to S. Danckwardt). Work in the lab of S.Danckwardt is supported by the EU 10.13039/501100007601Horizon 2020 Innovative training network ‘Thromboinflammation in Cardiovascular disorders’ (TICARDIO, Marie Skłodowska-Curie grant agreement No 813409). The work was also supported by the 10.13039/501100000921COST Action CA20121 – BenBedPhar and the environmental network EXPOHEALTH funded by the Science Initiative of the state Rhineland-Palatinate, Germany.

## Data Availability

Data will be made available on request.
