# Supplementary material for: Differential inflammation, oxidative stress and cardiovascular damage markers of nano- and micro-particle exposure in mice: Implications for human disease burden
Source: Redox Biol. 2025 Apr 22;83:103644. doi: 10.1016/j.redox.2025.103644 (PMC12124686; doi:10.1016/j.redox.2025.103644)
Supplement: Multimedia component 1 [file mmc1.pdf]

Online supplement

Differential inflammation, oxidative stress and cardiovascular damage markers of nano- and micro-particle exposure in mice: Implications for human disease burden

Marin Kuntic[1,2], Ivana Kuntic[1], Dirk Cleppien[3], Andrea Pozzer[4], David Nußbaum[1], Matthias Oelze[1], Tristan Junglas[1], Lea Strohm[1], Henning Ubbens[1], Steffen Daub[1], Maria Teresa Bayo Jimenez[1], Sven Danckwardt[2,5,6], Thomas Berkemeier[7], Omar Hahad[1,2], Matthias Kohl[4], Sebastian Steven[1,5,8], Albrecht Stroh[3,9], Jos Lelieveld[4], Thomas Münzel[1,2]*, Andreas Daiber[1,2,5]*

[1] University Medical Center Mainz, Department for Cardiology 1, Molecular Cardiology, Mainz, Germany

[2] German Center for Cardiovascular Research (DZHK), Partner Site Rhine-Main, Mainz, Germany

[3] Leibniz-Institut für Resilienzforschung (LIR) gGmbH, Mainz Animal Imaging Center (MAIC), Mainz, Germany

[4] Max Planck Institute for Chemistry, Atmospheric Chemistry Department, Mainz, Germany

[5] Center for Thrombosis and Hemostasis (CTH), University Medical Center of the Johannes Gutenberg-University, Mainz, Germany

[6] University Medical Center Ulm, Department of Clinical Chemistry, Ulm, Germany

[7] Max Planck Institute for Chemistry, Multiphase Chemistry Department, Mainz, Germany

[8] Division of Cardiology, Goethe University Frankfurt, University Hospital, Department of Medicine III, Frankfurt a. M., Germany

9 University Medical Center Mainz, Institute of Pathophysiology, Mainz, Germany

* These authors contributed equally and are joint senior authors.

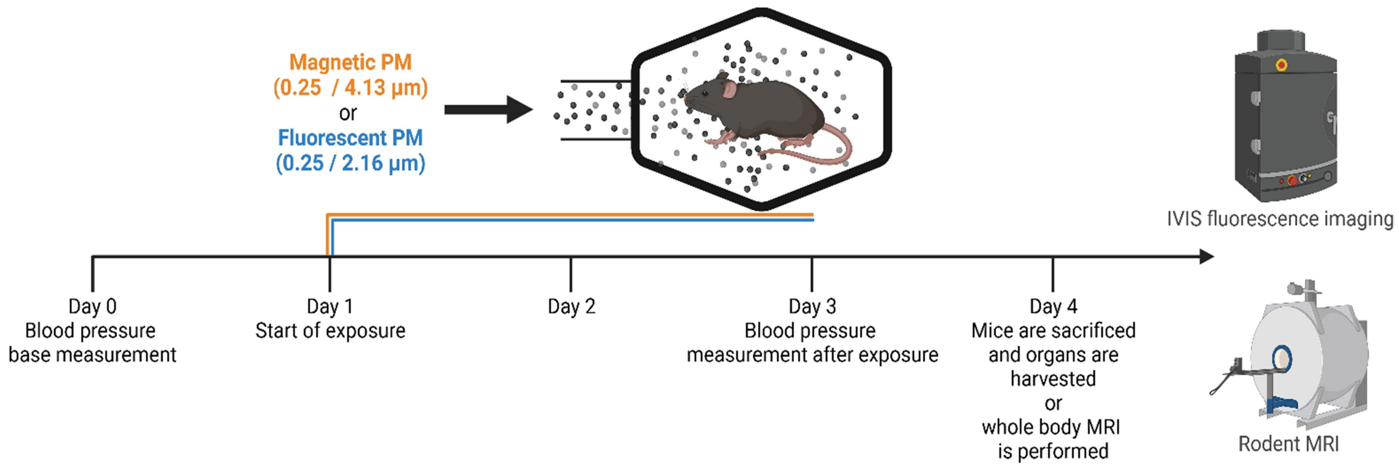

Suppl. Figure S1. Mouse exposure scheme. Mice were exposed to SPM for 3 consecutive days (start afternoon on day 1 and end morning on day 4), after which mice were either sacrificed and their organs were harvested for further experiments, including fluorescence imaging, or they were subjected to rodent magnetic resonance imaging. Blood pressure was measured at the baseline and after the final exposure. Created with Biorender.com

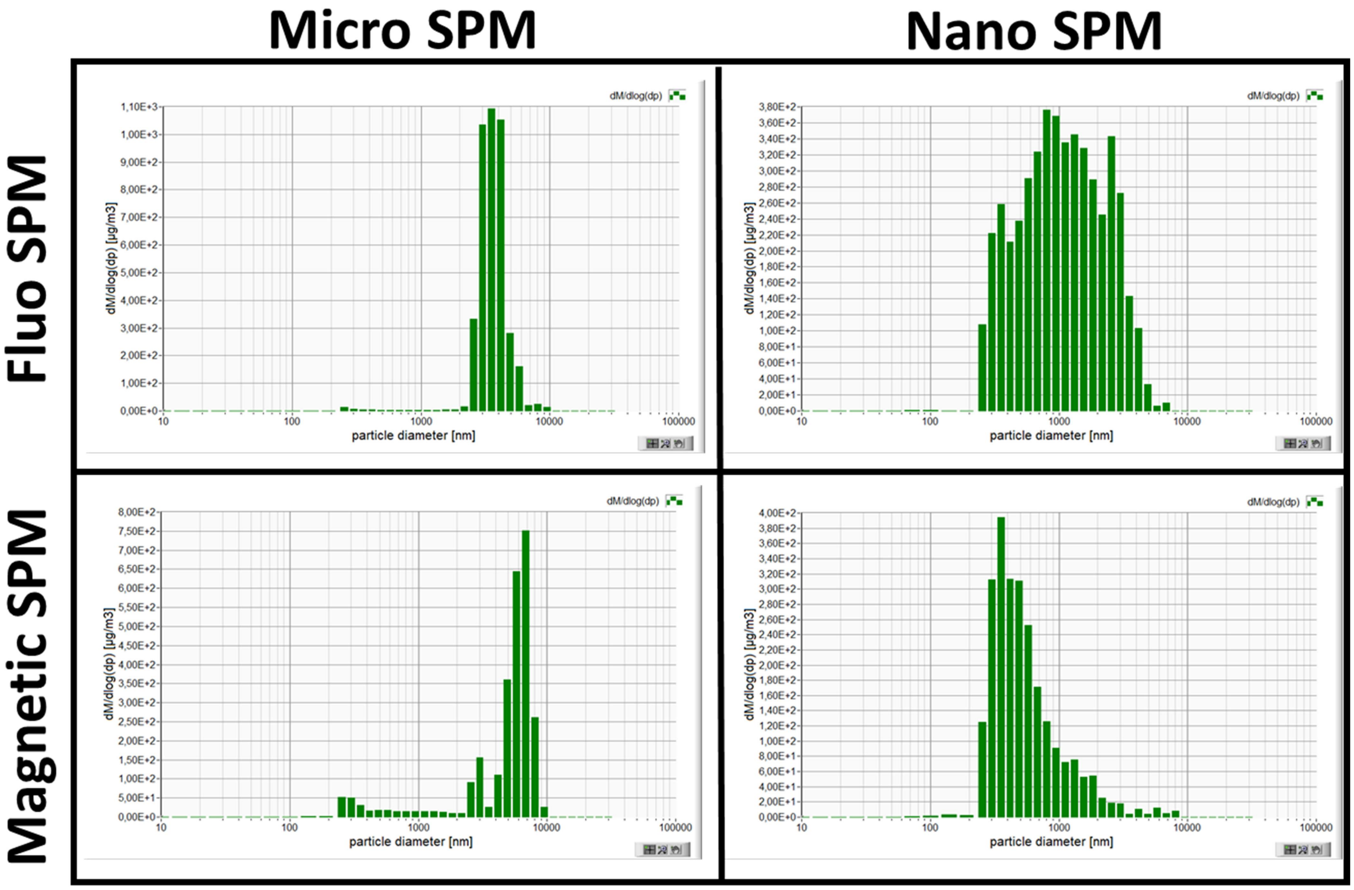

Suppl. Figure S2. Particle size distribution. Representative distributions for all of the different SPM materials used for the experiment, presented as mass distributions. Original diagrams generated by the NanoSpectroPan particle detector.

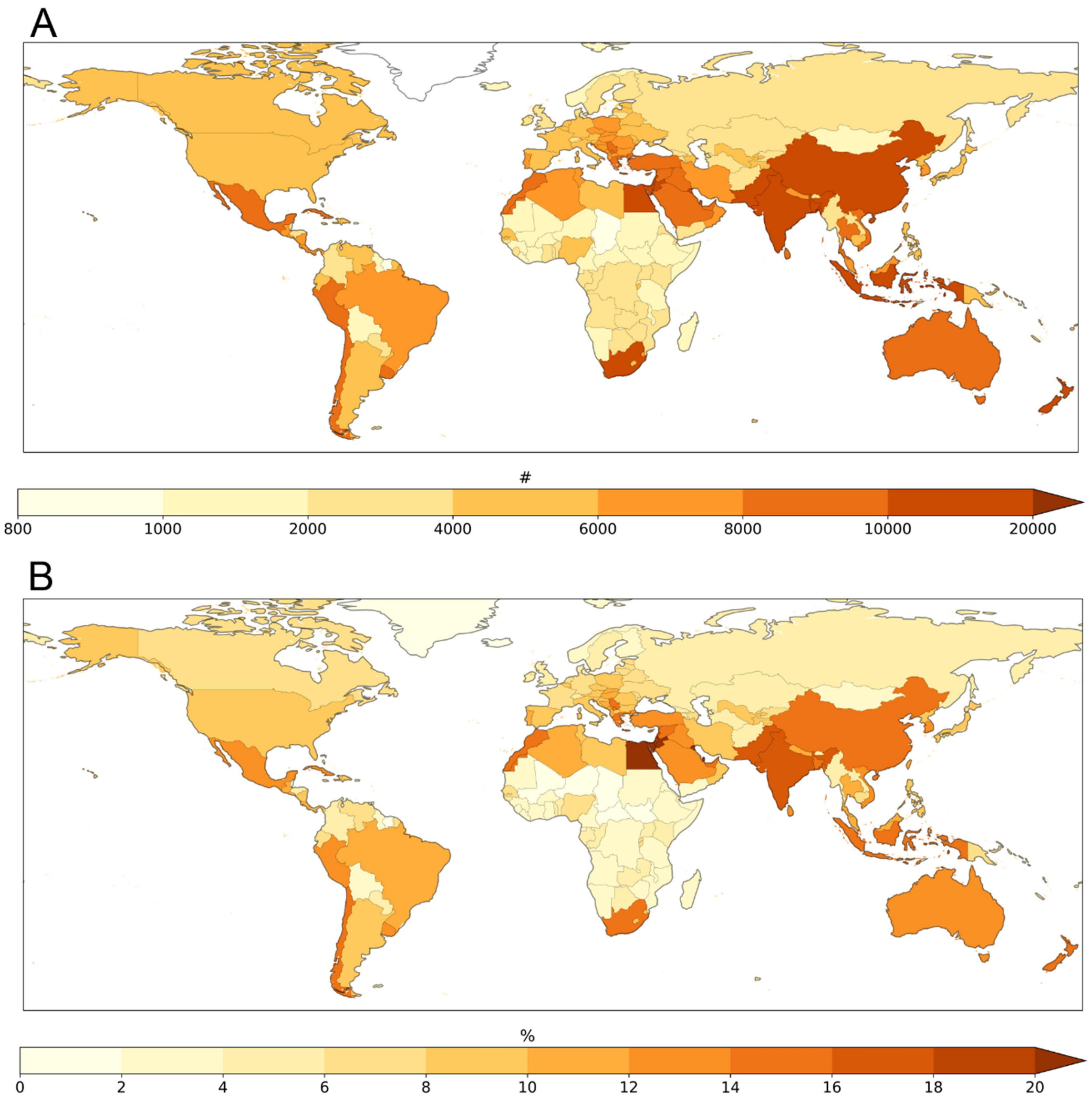

Suppl. Figure S3. Annual and country average, population-weighted exposure to UFP. Particle concentrations are in numbers per cm^3 (A). Country-average CVD incidence from UFP exposure relative to the total CVD incidence from all causes in percent (B).

Extended methods

Isometric tension studies in isolated aortic rings

Aortic ring segments from the thoracic part of the aorta, 4 mm in length, were cleaned from the perivascular adipose tissue and suspended from the force transducers in an organ bath [1, 2]. The force exerted by the aortic rings was measured in the presence of varying concentrations of vasodilators acetylcholine (ACh) and nitroglycerine (GTN) after preconstruction with prostaglandin F2α (yielding approximately 80 % of the maximal tone induced by KCl bolus). Endothelial function was determined by the addition of endothelium-dependent vasodilator, ACh in the range of 10^{-9} to $10^{-5.5}$ M. Endothelium-independent vasodilation was assessed by titrating the pre-constricted aortic rings with GTN in the range of 10^{-9} to $10^{-4.5}$ M. A constant temperature of 37 °C and flow of carbogen gas (95% oxygen, 5% CO_2 v/v) was maintained in the organ chamber throughout the experiment. The cyclooxygenase inhibitor indomethacin (10 µM) was added to the buffer to prevent the production of prostaglandins and other vasoactive eicosanoids that might interfere with the measurement.

Non-invasive blood pressure measurement

Blood pressure was measured using tail-cuff plethysmography with a blood pressure measurement instrument CODA (Kent Scientific, Torrington, CT) [1, 2]. Before the measurement, mice were restrained inside a plastic tube and placed on a preheated plate (32 °C). After a 15-minute rest two cuffs (occlusion cuff and volume pressure recording cuff) were placed on the tail of each mouse. The instrument performs 10 measurement iterations, and the mean is reported. Before the baseline blood pressure measurement was recorded, mice were trained without recording the results at least two times. The final experimental blood pressure (T1) value was recorded after the last exposure to PM.

Detection of fluorescent SPM in the isolated organs

After the exposure to fluorescent SPM, mice were sacrificed as described above and organs were excised. Removed organs were then imaged using the IVIS® Spectrum imaging system (PerkinElmer Inc, Waltham, MA, US) [3, 4]. For nano SPM excitation filter at 500 nm and emission filter at 560 nm was used, and for micro SPM excitation filter at 570 nm and emission filter at 620 nm was used. The mean pixels intensity from the images was obtained with the ImageJ software and used in the statistical analysis.

Detection of iron oxide SPM in the whole body via MRI

After the exposure to iron oxide SPM, mice were sacrificed by isoflurane overdose and whole bodies were frozen at -80°C. Before the magnetic resonance imaging (MRI) measurement, depicting the signal decrease caused by changes in T_2^* related to accumulated iron particles in the investigated tissue [5, 6], mice were heated to 25°C and kept at that temperature through the measurement by using a rectal temperature probe coupled with a ventilation system to maintain body temperature and avoid T_2^* drifts caused by temperature changes (Model 1030, Small Animal Instruments Inc., Stony Brook, NY, USA). A 9.4 T small animal MRI system with a 0.7 T/m gradient system (Biospec 94/20, Bruker Biospin GmbH, Ettlingen, Germany) controlled by Paravision 6.0.1 software was used for the measurements. To image the abdomen of the mice, a linear whole-body volume transmitter coil combined with an anatomically shaped 4-channel receive-only coil array for the rat brain was used. A 3D multigradient echo pulse sequence (TE/ TR = (3.5/ 800) ms; average = 2; flip angle = 50°; TA = 1 h 9 min) was carried out, recording 9 echoes with echo spacing = 5 ms to visualize the T_2^* signal decay. Isotropic voxels were measured with resolution of (0.25 x 0.25 x 0.25) mm^3 resulting in a 192 x 128 x 27 volume covering a FOV of 48 x 32 x 6.75 mm^3. The R_2^* parameter value per voxel was calculated by fitting an exponential decay curve to the corresponding pixels resulting in a volume of R_2^* values (MATLAB, R2022a; MathWorks; Natick, Massachusetts, USA). The R_2^* relaxation values were used for quantification, as the iron oxide SPM function as contrast agents and lower the T_2^* in the tissue they are present in.

Dihydroethidium fluorescence microtopography

Aortic sections from the thoracic part, cortex pieces and lung pieces were embedded in optimal cutting temperature (OCT) compound (TissueTek™, Sakura Finetek, Umkirch, Germany) and snap frozen in liquid nitrogen [7, 8]. The frozen blocks containing tissue pieces were cut on a cryo-microtome at -25 °C and thickness of 8 µm, transferred onto SuperFrost® (VWR International, Darmstadt, Germany) microscopy slides and stored at -80 °C. The tissue-containing slides were incubated with 1 µM dihydroethidium (DHE) for 30 minutes at 37 °C, washed twice with PBS, protected with a cover slide, and imaged under a fluorescence microscope (Axiovert 40CFL with Axiocam MRm, Zeiss, Germany). The excitation wavelength was set to 510 – 520 nm, and red fluorescence was recorded (emission: 580 – 610 nm). The fluorescence images were quantified as the mean pixel intensity obtained from the area of interest (endothelium and media for the aorta and whole image for lung and cortex) using ImageJ software.

Western blot analysis

Protein expression in tissues of interest was determined by a standard western blot analysis [2, 9]. Protein samples were analyzed using specific primary antibodies against endothelial NO-synthase (eNOS, 1:1000, BD Bioscience #610297, USA), endothelin-1 (ET-1, 1:1000, mouse monoclonal, SantaCruz #sc-517436, Dallas, USA), NADPH oxidase subunits gp91phox (NOX2, mouse monoclonal gp91phox, 1:500, BD Biosciences #611415, USA)), NOX1 (rabbit polyclonal, 1:500, Abcam #ab131088, Cambridge, MA, USA), p67phox (1:1000, BD Bioscience #610913, USA) and phosphorylated p47phox (Neutrophil Cytosolic Factor 1 (NCF-1), 1:000, AssayBiotech #A1161, Sunnyvale, CA), myristoylated alanine-rich protein kinase C substrate phosphorylated at Ser152/156 (P-MARCKS, 1:1000, Cell Signaling, Danvers, MA), cluster of differentiation 68 (CD68, 1:1000, Abcam #ab31630, Cambridge, MA, USA), Manganese superoxide dismutase (MnSOD (SOD-2), 1:1000, Millipore #06-984, Lake Placid, NY), protein kinase Cα1 (PKCα1, 1:5000, BD Bioscience #610107, USA), monocyte chemoattractant protein 1 (MCP-1, 0.4 µg/mL, BioRad #AAM43, Feldkirchen, Germany), heme oxygenase 1 (HO-1, 1:250, Abcam #ab68477, Cambridge, MA, USA), dihydrofolate reductase (DHFR, 1:500, Novus Biologicals, Littleton, CO), and α-actinin or β-actin (1:2500 each, Sigma-Aldrich #A5044 and #A5060, St. Louis, MO) for normalization against loading and transfer. Horseradish-peroxidase conjugated anti-mouse or anti-rabbit were used as secondary antibodies

(1:10000 each, Vector Lab. #PI-2000 (anti-mouse IgG) and #PI-1000 (anti-rabbit IgG), Burlingame, CA). Densitometric quantification of antibody-specific bands was performed with an ECL Chemostar Imager (Intas Science Imaging Instruments GmbH, Germany) and Gel-Pro Analyzer software.

Modeling atmospheric exposure

We applied a data-informed global atmospheric modelling method to compute the exposure to air pollutants. The Earth system model for atmospheric chemistry and climate (EMAC) used in this study was applied at a horizontal resolution of about 1.875° latitude and longitude, with 31 vertical levels up to 10 hPa (~30 km altitude) [10, 11]. The anthropogenic emissions of trace gases and particles used as model input have been adopted from the Community Emission Data System (CEDS) [12]. Source sectors include fossil energy production, industry, land transport, shipping, aviation, domestic energy use from solid biofuels, waste incineration, agriculture, solvent production and use. The CEDS emission data have been produced at a geographical resolution of 0.5°. In addition, we employed the Emissions Database for Global Atmospheric Research (EDGAR [13]) at 0.1° latitude and longitude for downscaling to higher resolution. The emission size spectrum of aerosol particles depends on the source sectors, according to published work [14], and has been optimized according to size distribution measurements [15]. A comprehensive evaluation of the modelled atmospheric dust, black and organic carbon, aerosol optical depth, and aerosol organic and inorganic compounds was presented previously [16].

Downscaling of UFP concentrations

Since the relatively coarse grid resolution of the global EMAC model does not do justice to concentration gradients near strong sources of ultrafine particles (UFP), in particular of primary particles near areas with heavy traffic and industrial emission hotspots, we downscaled the simulation results in two steps, first to 0.5° and then to 0.1° latitude and longitude (~9 km at mid-latitudes). Observation-guided downscaling was achieved by redistributing the model grid box average UFP concentration according to the anthropogenic source sectors, available at higher resolution in the CEDS and EDGAR emission inventories [15]. UFP

results were evaluated against the measured number of particles in the size fraction 3 nm to 100 nm, the former based on the lower size limit of the measurements. A linear relationship between the observed and modelled particle number concentrations was applied to redistribute the particles toward the source areas, successively in the 0.5° and 0.1° inventories. Comparison of the results with long-term measured UFP concentrations at 60 measurement locations in Europe, India, China, North America and remote locations worldwide indicate good agreement [15]. The logarithmic correlation coefficient is r = 0.95 (the linear r = 0.99), the slope of the linear fit is 1.022, and the root mean square log error is 0.43.

Estimation of health impacts

Incident cardiovascular disease (CVD), *C*, attributable to the long-term exposure to UFP at geographical coordinates *x* and *y*, *M* (*x,y*), was calculated by:

$$M(x,y) = \sum_j AF_j[X(x,y)] \cdot C_j(x,y) \cdot P(x,y)$$

where *j* refers to the age category >25 years, and *X* is the concentration of UFP [17]. *AF* is the attributable fraction of the CVD incidence due to exposure, and *P* is the population at the geographical coordinates, i.e., the 0.1° grid cells for which we computed UFP exposure. The incident CVD and population data have been adopted from the Global Burden of Disease [18]. The *AF* has been derived from the hazard ratio (*HR*) associated with exposure to UFP from data of a published cohort study [19], which identified the increased risk of all incident CVD:

$$AF_j = (HR_j(UFP) - 1)/HR_j(UFP)$$

A log-normal exposure-response function was applied to describe the dependency of *HR* on the concentration of UFP, according to $HR_j = exp\,(\beta \times X)$. The factor β was estimated from previous results [19] by using an *HR* of 1.18 (95% confidence interval (CI): 1.03–1.34) per 10,000 particles cm^{-3}, obtaining $\beta = 1.6 \times 10^{-5}$ (95% CI: $2.9\times10^{-6} - 2.9\times10^{-5}$). Note that we did not apply a theoretical minimum risk exposure level – whether there is one is unknown. The 95% confidence intervals in all our results have been derived by adopting the ranges as previously described [19].

Extended results

Global exposure to UFP

Potential cardiovascular health impacts of ambient levels of UFP were also studied by combining regional and global exposure data with results from the only epidemiological cohort study currently available [20]. **Figure S3A** presents the country-level, mean, and population-weighted exposure to UFP, aggregated from the downscaled data available at a spatial resolution of 0.1° latitude and longitude. The period considered is one year for which coincident emission inventories and measurement data were available, based on recently published results [15]. We concentrate on long-term –rather than acute– exposure because chronic oxidative stress and inflammatory responses are associated with cardiovascular, cardiometabolic, and cerebrovascular diseases [21]. We find that countries with the highest exposure are found in the Middle East and parts of Asia, largely associated with the urban environment. Annual and country mean concentrations reach up to 20,000 particles cm^{-3}, e.g., in Singapore, Arabian Gulf states, and Egypt, and somewhat lower (up to 11,000 cm^{-3}) in other Middle Eastern countries (e.g., Israel, Jordan), and South and East Asia. Exposure is also very high in South Africa, New Zealand, Australia, Mexico, and several South American, North African, and Eastern European countries. In Europe, country and annual mean UFP exposure ranges from about 2,000–3,000 cm^{-3} in Scandinavia, 10,000 cm^{-3} in Balkan states, and 4,000–6,000 cm^{-3} in Western and Central Europe.

Figure S3B shows these estimated fractions for all countries worldwide, suggesting particularly relevant implications for CVD incidence from UFP exposure in Middle Eastern and South Asian countries, for example. These results should be considered preliminary, i.e., first-order estimates until additional epidemiological studies become available that account for air pollution, including UFP, and health conditions in representative regions of the world. The annual global mean per capita CVD incidence due to UFP is estimated at 76.2 (95% UI: 16.2–115.2) per 100,000 population. CVD is the globally leading cause of death, amounting to 18.6 (95% UI: 17.1-19.7) million per year, nearly one-third of the all-cause mortality [22]. By further hypothesising that CVD incidence is proportional to CVD mortality, we estimate that 2.0 (95% UI: 0.4–3.3) million CVD-related deaths per year could potentially be attributable to UFP exposure.

We reiterate that our estimates should be considered preliminary, but they underscore the major public health challenges associated with chronic exposure to UFP in polluted air. The recent global CVD prevalence is about 0.5 billion cases, following an upsurge of about 50% in the past three decades, particularly outside high-income countries [22]. This is remarkably coincident with the strongly increasing levels of air pollution in low- and middle-income countries, whereas high- (and some middle-) income countries have generally improved air quality [23]. Regardless of the uncertainties, UFP appears to contribute significantly to cardiovascular morbidity and, consequently, associated mortality. The predominant anthropogenic source of exposure is the use of fossil fuels in energy generation, industry, and traffic, indicating that a transition to renewable, non-combustion energy sources could have major cardiovascular health benefits.

Extended limitations of the study

During the study, we noticed several limitations regarding PM exposure studies in general and the current exposure protocol in particular. One of the major hurdles in assessing the effects of PM with different sizes is the concentration and size distribution. As most of the PM is not uniform in size but follows a certain distribution (e.g. Gaussian), it is quite challenging to achieve a precise exposure concentration (mass flow in $\mu g/m^3$ or particle flow in N/m^3). The difficulty arises because larger particles carry disproportionately more mass than small particles, and mass concentration is the usual way of defining PM concentration, making it highly dependent on the larger particles (mass distribution showed in **Figure S2**). The opposite is true for the number distribution, as smaller particles are more abundant for the same total mass. Whenever a size distribution of PM exists, it is expected that small PM will accompany PM of a larger average diameter and that large PM will significantly contribute to mass concentration, whereas the particle number is largely based on PM with a smaller average diameter. Therefore, it is difficult to draw conclusions about PM health effects based solely on average PM diameter, especially in light of different penetrating depths and organ distribution.

In our experiments, the low amount of PM available through inhalation was insufficient to produce a clear contrast in the MRI experiments. The amount of PM that entered the mouse through inhalation, calculated to be approximately 40 – 120 µg/kg, was at least one order of magnitude lower than the lowest literature values for human contrast applications, where the lower limit is roughly 1000 µg/kg [24, 25]. In addition, this approximated amount of PM is calculated for a whole 6-hour exposure session per day, and the clearance, which can vary greatly, was not taken into account [26]. The larger amount of PM needed for an optimal MRI contrast would require an unrealistically high exposure concentration of more than 2000 $\mu g/m^3$, leading to questionable exposure conditions regarding real-world scenarios. The only way this could be mitigated in the future is through longer exposure time, resulting in the potential accumulation of magnetic particles in the liver, heart, spleen, or brain, where it is taken up by the resident macrophages [27]. However, there are high uncertainties due to not well-characterized clearance processes in healthy mice and differences in particle coating (silica vs. PEG vs. polystyrene), as some studies imply fast clearance in a matter of days [28, 29], and others have observed almost no clearance after as long as 28 days [30] or even 6 months [31].

We attributed the absence of the accumulation of fluorescent nano-sized SPM in the lung of exposed mice to their transmigration into the circulation. However, because exhaled particles were not measured in the present study, the lung's lack of a pronounced fluorescence signal could be due to the reported higher exhalation rate of nanoparticles [32]. Despite this limitation and uncertainty, the assumption of transmigration of nano-sized SPM through the lung was further supported by the present observation of an accumulation of magnetic nanoparticles in the liver by trend. Also, the more pronounced effects of nano-sized SPM compared to microparticles on functional parameters, e.g. blood pressure increase, and oxidative stress parameters and markers of inflammation in remote organs such as the aorta, heart and brain point towards more efficient transmigration of nanoparticles through the lung. These assumptions are also in accordance with human data on the association of UFP exposure with cardiovascular but not respiratory disease risk [33-35] and reported direct effects of nanoparticles on the brain of mice and humans [36-38].

The DHE staining performed in this study to analyze the ROS production is not an in-depth analysis of oxidative stress, but rather a surrogate marker of the ROS levels. For an in-depth analysis of the redox state of a cell or whether oxidative stress conditions are present, both ROS producing enzymes and individual ROS should be determined, preferential together with antioxidants and some oxidative posttranslational protein modifications as well [39]. DHE staining imaged with florescence microscopy provides only a cumulative measurement of H_2O_2 and $O_2^{\cdot -}$ production (since the two oxidation products, 2-hydroxyethidium and ethidium cannot be distinguished) without taking into account the compensatory mechanisms of redox homeostasis.

Extended references

[1] S. Kroller-Schon, A. Daiber, S. Steven, M. Oelze, K. Frenis, S. Kalinovic, A. Heimann, F.P. Schmidt, A. Pinto, M. Kvandova, K. Vujacic-Mirski, K. Filippou, M. Dudek, M. Bosmann, M. Klein, T. Bopp, O. Hahad, P.S. Wild, K. Frauenknecht, A. Methner, E.R. Schmidt, S. Rapp, H. Mollnau, T. Munzel. Crucial role for Nox2 and sleep deprivation in aircraft noise-induced vascular and cerebral oxidative stress, inflammation, and gene regulation. Eur Heart J 39 (2018) 3528-3539, https://doi.org/10.1093/eurheartj/ehy333.

[2] T. Munzel, A. Daiber, S. Steven, L.P. Tran, E. Ullmann, S. Kossmann, F.P. Schmidt, M. Oelze, N. Xia, H. Li, A. Pinto, P. Wild, K. Pies, E.R. Schmidt, S. Rapp, S. Kroller-Schon. Effects of noise on vascular function, oxidative stress, and inflammation: mechanistic insight from studies in mice. Eur Heart J 38 (2017) 2838-2849, https://doi.org/10.1093/eurheartj/ehx081.

[3] S. Steven, K. Jurk, M. Kopp, S. Kroller-Schon, Y. Mikhed, K. Schwierczek, S. Roohani, F. Kashani, M. Oelze, T. Klein, S. Tokalov, S. Danckwardt, S. Strand, P. Wenzel, T. Munzel, A. Daiber. Glucagon-like peptide-1 receptor signalling reduces microvascular thrombosis, nitro-oxidative stress and platelet activation in endotoxaemic mice. Br J Pharmacol 174 (2017) 1620-1632, https://doi.org/10.1111/bph.13549.

[4] S.V. Tokalov, D. Bachiller. IV delivery of fluorescent beads. Chest 141 (2012) 833-834, https://doi.org/10.1378/chest.11-2695.

[5] A. Stroh, C. Faber, T. Neuberger, P. Lorenz, K. Sieland, P.M. Jakob, A. Webb, H. Pilgrimm, R. Schober, E.E. Pohl, C. Zimmer. In vivo detection limits of magnetically labeled embryonic stem cells in the rat brain using high-field (17.6 T) magnetic resonance imaging. Neuroimage 24 (2005) 635-645, https://doi.org/10.1016/j.neuroimage.2004.09.014.

[6] A. Stroh, C. Zimmer, N. Werner, K. Gertz, K. Weir, G. Kronenberg, J. Steinbrink, S. Mueller, K. Sieland, U. Dirnagl, G. Nickenig, M. Endres. Tracking of systemically administered mononuclear cells in the ischemic brain by high-field magnetic resonance imaging. Neuroimage 33 (2006) 886-897, https://doi.org/10.1016/j.neuroimage.2006.07.009.

[7] M. Kuntic, I. Kuntic, R. Krishnankutty, A. Gericke, M. Oelze, T. Junglas, M.T. Bayo Jimenez, P. Stamm, M. Nandudu, O. Hahad, K. Keppeler, S. Daub, K. Vujacic-Mirski, S. Rajlic, L. Strohm, H. Ubbens, Q. Tang, S. Jiang, Y. Ruan, K.G. Macleod, S. Steven, T. Berkemeier, U. Poschl, J. Lelieveld, H. Kleinert, A. von Kriegsheim, A. Daiber, T. Munzel. Co-exposure to urban particulate matter and aircraft noise adversely impacts the cerebro-pulmonary-cardiovascular axis in mice. Redox Biol 59 (2023) 102580, https://doi.org/10.1016/j.redox.2022.102580.

[8] M. Kuntic, M. Oelze, S. Steven, S. Kroller-Schon, P. Stamm, S. Kalinovic, K. Frenis, K. Vujacic-Mirski, M.T. Bayo Jimenez, M. Kvandova, K. Filippou, A. Al Zuabi, V. Bruckl, O. Hahad, S. Daub, F. Varveri, T. Gori, R. Huesmann, T. Hoffmann, F.P. Schmidt, J.F. Keaney, A. Daiber, T. Munzel. Short-term e-cigarette vapour exposure causes vascular oxidative stress and dysfunction: evidence for a close connection to brain damage and a key role of the phagocytic NADPH oxidase (NOX-2). Eur Heart J 41 (2020) 2472-2483, https://doi.org/10.1093/eurheartj/ehz772.

[9] J. Renart, J. Reiser, G.R. Stark. Transfer of proteins from gels to diazobenzyloxymethyl-paper and detection with antisera: a method for studying antibody specificity and antigen structure. Proc Natl Acad Sci U S A 76 (1979) 3116-3120, https://doi.org/10.1073/pnas.76.7.3116.

[10] P. Jöckel, H. Tost, A. Pozzer, e. al. Earth System Chemistry integrated Modelling (ESCiMo) with the Modular Earth Submodel System (MESSy) version 2.51. . Geoscientific Model Development 9 (2016) 1153-1200.

[11] A. Pozzer, S.F. Reifenberg, V. Kumar, e. al. Simulation of organics in the atmosphere: evaluation of EMACv2.54 with the Mainz Organic Mechanism (MOM) coupled to the ORACLE (v1.0) submodel. Geoscientific Model Development 15 (2022) 2673-2710.

[12] E.E. McDuffie, S.J. Smith, P. O'Rourke, K. Tibrewal, C. Venkataraman, E.A. Marais, B. Zheng, M. Crippa, M. Brauer, R.V. Martin. A global anthropogenic emission inventory of atmospheric pollutants from sector- and fuel-specific sources (1970–2017): an application of the Community Emissions Data System (CEDS). Earth System Science Data 12 (2020) 3413–3442, https://doi.org/https://doi.org/10.5194/essd-12-3413-2020.

[13] M. Crippa, E. Solazzo, G. Huang, D. Guizzardi, E. Koffi, M. Muntean, C. Schieberle, R. Friedrich, G. Janssens-Maenhout. High resolution temporal profiles in the Emissions Database for Global Atmospheric Research. Sci Data 7 (2020) 121, https://doi.org/10.1038/s41597-020-0462-2.

[14] P. Paasonen, K. Kupiainen, Z. Klimont, A. Visschedijk, H.A.C.D. van der Gon, M. Amann. Continental anthropogenic primary particle number emissions. . Atmospheric Chemistry and Physics 16 (2016) 6823-6840.

[15] M. Kohl, J. Lelieveld, S. Chowdhury, S. Ehrhart, D. Sharma, Y. Cheng, S.N. Tripathi, M. Sebastian, G. Pandithurai, H. Wang, A. Pozzer. Numerical simulation and evaluation of global ultrafine particle concentrations at the Earth's surface. Atmos. Chem. Phys. 23 (2023) 13191–13215, https://doi.org/https://doi.org/10.5194/acp-23-13191-2023.

[16] A. Pozzer, S.F. Reifenberg, V. Kumar, B. Franco, M. Kohl, D. Taraborrelli, S. Gromov, S. Ehrhart, P. Jöckel, R. Sander, V. Fall, S. Rosanka, V. Karydis, D. Akritidis, T. Emmerichs, M. Crippa, D. Guizzardi, J.W. Kaiser, L. Clarisse, A. Kiendler-Scharr, H. Tost, A. Tsimpidi. Simulation of organics in the atmosphere: evaluation of EMACv2.54 with the Mainz Organic Mechanism (MOM) coupled to the ORACLE (v1.0) submodel. Geoscientific Model Development 15 (2022) 2673-2710, https://doi.org/10.5194/gmd-15-2673-2022.

[17] A. Pozzer, S.C. Anenberg, S. Dey, A. Haines, J. Lelieveld, S. Chowdhury. Mortality Attributable to Ambient Air Pollution: A Review of Global Estimates. Geohealth 7 (2023), https://doi.org/ARTN e2022GH000711

10.1029/2022GH000711.

[18] G.B.D.R.F. Collaborators. Global burden of 87 risk factors in 204 countries and territories, 1990-2019: a systematic analysis for the Global Burden of Disease Study 2019. Lancet 396 (2020) 1223-1249, https://doi.org/10.1016/S0140-6736(20)30752-2.

[19] G.S. Downward, E.J.H.M. van Nunen, J. Kerckhoffs, P. Vineis, B. Brunekreef, J.M.A. Boer, K.P. Messier, A. Roy, W.M.M. Verschuren, Y.T. van der Schouw, I. Sluijs, J. Gulliver, G. Hoek, R. Vermeulen. Long-Term Exposure to Ultrafine Particles and Incidence of Cardiovascular and Cerebrovascular Disease in a Prospective Study of a Dutch Cohort. Environmental Health Perspectives 126 (2018), https://doi.org/Artn 127007

10.1289/Ehp3047.

[20] G.S. Downward, E. van Nunen, J. Kerckhoffs, P. Vineis, B. Brunekreef, J.M.A. Boer, K.P. Messier, A. Roy, W.M.M. Verschuren, Y.T. van der Schouw, I. Sluijs, J. Gulliver, G. Hoek, R. Vermeulen. Long-Term Exposure to Ultrafine Particles and Incidence of Cardiovascular and Cerebrovascular Disease in a Prospective Study of a Dutch Cohort. Environ Health Perspect 126 (2018) 127007, https://doi.org/10.1289/EHP3047.

[21] A. Peters, T.S. Nawrot, A.A. Baccarelli. Hallmarks of environmental insults. Cell 184 (2021) 1455-1468, https://doi.org/10.1016/j.cell.2021.01.043.

[22] G.A. Roth, G.A. Mensah, C.O. Johnson, G. Addolorato, E. Ammirati, L.M. Baddour, N.C. Barengo, A.Z. Beaton, E.J. Benjamin, C.P. Benziger, A. Bonny, M. Brauer, M. Brodmann, T.J. Cahill, J. Carapetis, A.L. Catapano, S.S.

Chugh, L.T. Cooper, J. Coresh, M. Criqui, N. DeCleene, K.A. Eagle, S. Emmons-Bell, V.L. Feigin, J. Fernandez-Sola, G. Fowkes, E. Gakidou, S.M. Grundy, F.J. He, G. Howard, F. Hu, L. Inker, G. Karthikeyan, N. Kassebaum, W. Koroshetz, C. Lavie, D. Lloyd-Jones, H.S. Lu, A. Mirijello, A.M. Temesgen, A. Mokdad, A.E. Moran, P. Muntner, J. Narula, B. Neal, M. Ntsekhe, G. Moraes de Oliveira, C. Otto, M. Owolabi, M. Pratt, S. Rajagopalan, M. Reitsma, A.L.P. Ribeiro, N. Rigotti, A. Rodgers, C. Sable, S. Shakil, K. Sliwa-Hahnle, B. Stark, J. Sundstrom, P. Timpel, I.M. Tleyjeh, M. Valgimigli, T. Vos, P.K. Whelton, M. Yacoub, L. Zuhlke, C. Murray, V. Fuster, G.-N.-J.G.B.o.C.D.W. Group. Global Burden of Cardiovascular Diseases and Risk Factors, 1990-2019: Update From the GBD 2019 Study. J Am Coll Cardiol 76 (2020) 2982-3021, https://doi.org/10.1016/j.jacc.2020.11.010.

[23] P. Hystad, A. Larkin, S. Rangarajan, K.F. AlHabib, A. Avezum, K.B.T. Calik, J. Chifamba, A. Dans, R. Diaz, J.L. du Plessis, R. Gupta, R. Iqbal, R. Khatib, R. Kelishadi, F. Lanas, Z. Liu, P. Lopez-Jaramillo, S. Nair, P. Poirier, O. Rahman, A. Rosengren, H. Swidan, L.A. Tse, L. Wei, A. Wielgosz, K. Yeates, K. Yusoff, T. Zatonski, R. Burnett, S. Yusuf, M. Brauer. Associations of outdoor fine particulate air pollution and cardiovascular disease in 157 436 individuals from 21 high-income, middle-income, and low-income countries (PURE): a prospective cohort study. Lancet Planet Health 4 (2020) e235-e245, https://doi.org/10.1016/S2542-5196(20)30103-0.

[24] C.M. Colbert, Z. Ming, A. Pogosyan, J.P. Finn, K.L. Nguyen. Comparison of Three Ultrasmall, Superparamagnetic Iron Oxide Nanoparticles for MRI at 3.0 T. J Magn Reson Imaging 57 (2023) 1819-1829, https://doi.org/10.1002/jmri.28457.

[25] Y. Lu, J. Huang, N.V. Neverova, K.L. Nguyen. USPIOs as targeted contrast agents in cardiovascular magnetic resonance imaging. Curr Cardiovasc Imaging Rep 14 (2021), https://doi.org/10.1007/s12410-021-09552-8.

[26] P. Keselman, E.Y. Yu, X.Y. Zhou, P.W. Goodwill, P. Chandrasekharan, R.M. Ferguson, A.P. Khandhar, S.J. Kemp, K.M. Krishnan, B. Zheng, S.M. Conolly. Tracking short-term biodistribution and long-term clearance of SPIO tracers in magnetic particle imaging. Phys Med Biol 62 (2017) 3440-3453, https://doi.org/10.1088/1361-6560/aa5f48.

[27] H.H. Tan, M.I. Fiel, Q. Sun, J. Guo, R.E. Gordon, L.C. Chen, S.L. Friedman, J.A. Odin, J. Allina. Kupffer cell activation by ambient air particulate matter exposure may exacerbate non-alcoholic fatty liver disease. J Immunotoxicol 6 (2009) 266-275, https://doi.org/10.1080/15476910903241704.

[28] C. Zhou, M. Long, Y. Qin, X. Sun, J. Zheng. Luminescent gold nanoparticles with efficient renal clearance. Angew Chem Int Ed Engl 50 (2011) 3168-3172, https://doi.org/10.1002/anie.201007321.

[29] C. Alric, I. Miladi, D. Kryza, J. Taleb, F. Lux, R. Bazzi, C. Billotey, M. Janier, P. Perriat, S. Roux, O. Tillement. The biodistribution of gold nanoparticles designed for renal clearance. Nanoscale 5 (2013) 5930-5939, https://doi.org/10.1039/c3nr00012e.

[30] X. Li, B. Wang, S. Zhou, W. Chen, H. Chen, S. Liang, L. Zheng, H. Yu, R. Chu, M. Wang, Z. Chai, W. Feng. Surface chemistry governs the sub-organ transfer, clearance and toxicity of functional gold nanoparticles in the liver and kidney. J Nanobiotechnology 18 (2020) 45, https://doi.org/10.1186/s12951-020-00599-1.

[31] E. Sadauskas, G. Danscher, M. Stoltenberg, U. Vogel, A. Larsen, H. Wallin. Protracted elimination of gold nanoparticles from mouse liver. Nanomedicine 5 (2009) 162-169, https://doi.org/10.1016/j.nano.2008.11.002.

[32] I.M. El-Sherbiny, N.M. El-Baz, M.H. Yacoub. Inhaled nano- and microparticles for drug delivery. Glob Cardiol Sci Pract 2015 (2015) 2, https://doi.org/10.5339/gcsp.2015.2.

[33] X. Meng, Y. Ma, R. Chen, Z. Zhou, B. Chen, H. Kan. Size-fractionated particle number concentrations and daily mortality in a Chinese city. Environ Health Perspect 121 (2013) 1174-1178, https://doi.org/10.1289/ehp.1206398.

[34] M. Stolzel, S. Breitner, J. Cyrys, M. Pitz, G. Wolke, W. Kreyling, J. Heinrich, H.E. Wichmann, A. Peters. Daily mortality and particulate matter in different size classes in Erfurt, Germany. J Expo Sci Environ Epidemiol 17 (2007) 458-467, https://doi.org/10.1038/sj.jes.7500538.

[35] M.L. Bergmann, Z.J. Andersen, A. Massling, P.A. Kindler, S. Loft, H. Amini, T. Cole-Hunter, Y. Guo, M. Maric, C. Nordstrom, M. Taghavi, S. Tuffier, R. So, J. Zhang, Y.H. Lim. Short-term exposure to ultrafine particles and mortality and hospital admissions due to respiratory and cardiovascular diseases in Copenhagen, Denmark. Environ Pollut 336 (2023) 122396, https://doi.org/10.1016/j.envpol.2023.122396.

[36] H. Cheng, A. Saffari, C. Sioutas, H.J. Forman, T.E. Morgan, C.E. Finch. Nanoscale Particulate Matter from Urban Traffic Rapidly Induces Oxidative Stress and Inflammation in Olfactory Epithelium with Concomitant Effects on Brain. Environ Health Perspect 124 (2016) 1537-1546, https://doi.org/10.1289/EHP134.

[37] A. Gonzalez-Maciel, R. Reynoso-Robles, R. Torres-Jardon, P.S. Mukherjee, L. Calderon-Garciduenas. Combustion-Derived Nanoparticles in Key Brain Target Cells and Organelles in Young Urbanites: Culprit Hidden in Plain Sight in Alzheimer's Disease Development. J Alzheimers Dis 59 (2017) 189-208, https://doi.org/10.3233/JAD-170012.

[38] A. Haghani, R. Johnson, N. Safi, H. Zhang, M. Thorwald, A. Mousavi, N.C. Woodward, F. Shirmohammadi, V. Coussa, J.P. Wise, Jr., H.J. Forman, C. Sioutas, H. Allayee, T.E. Morgan, C.E. Finch. Toxicity of urban air pollution particulate matter in developing and adult mouse brain: Comparison of total and filter-eluted nanoparticles. Environ Int 136 (2020) 105510, https://doi.org/10.1016/j.envint.2020.105510.

[39] H. Sies, V.V. Belousov, N.S. Chandel, M.J. Davies, D.P. Jones, G.E. Mann, M.P. Murphy, M. Yamamoto, C. Winterbourn. Defining roles of specific reactive oxygen species (ROS) in cell biology and physiology. Nat Rev Mol Cell Biol 23 (2022) 499-515, https://doi.org/10.1038/s41580-022-00456-z.
